# Supplementary material for: P2RY14 cAMP signaling regulates Schwann cell precursor self-renewal, proliferation, and nerve tumor initiation in a mouse model of neurofibromatosis
Source: eLife. 2022 Mar 21;11:e73511. doi: 10.7554/eLife.73511 (PMC8959601; doi:10.7554/eLife.73511)

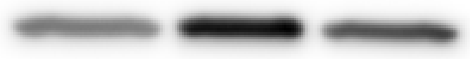

Supplement: Source data 1. [file elife-73511-data1.zip › Source data 1/Figure 5I b-actin-source data 1.pdf]
