# Supplementary material for: P2RY14 cAMP signaling regulates Schwann cell precursor self-renewal, proliferation, and nerve tumor initiation in a mouse model of neurofibromatosis
Source: eLife. 2022 Mar 21;11:e73511. doi: 10.7554/eLife.73511 (PMC8959601; doi:10.7554/eLife.73511)
Supplement: Source data 1. [file elife-73511-data1.zip › Source data 1/Figure 3G-2-source data 1.pdf]

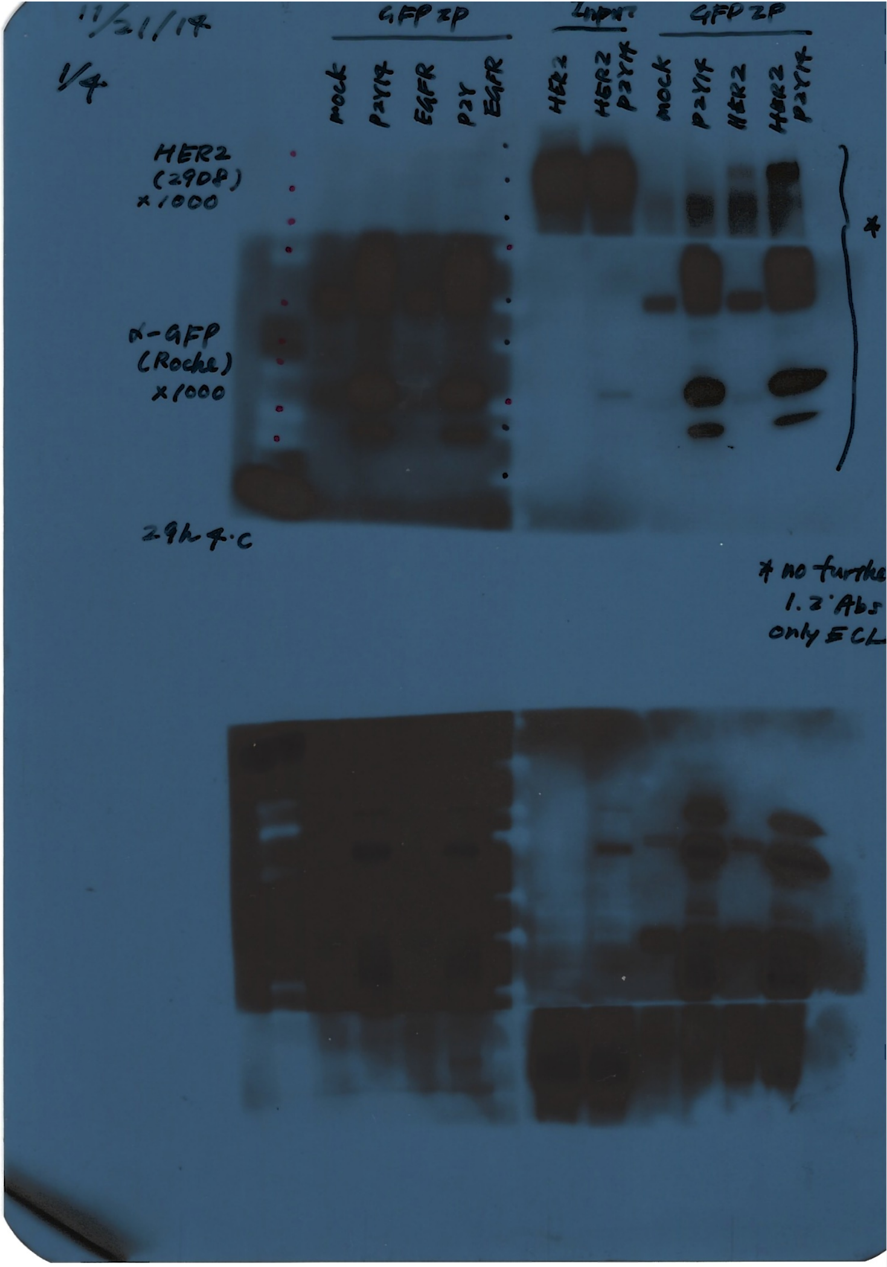
11/21/14
1/4
GFP IP
Input
GFP IP
mock
P2Y14
EGFR
P2Y EGFR
HER2
HER2 P2Y14
mock
P2Y14
HER2
HER2 P2Y14
HER2 (2908) x1000
α-GFP (Roche) x1000
29h 4°C
*
* no furthe
1.2°Abs
only ECL
