# Supplementary figures and images for: P2RY14 cAMP signaling regulates Schwann cell precursor self-renewal, proliferation, and nerve tumor initiation in a mouse model of neurofibromatosis
Source: eLife. 2022 Mar 21;11:e73511. doi: 10.7554/eLife.73511 (PMC8959601; doi:10.7554/eLife.73511)

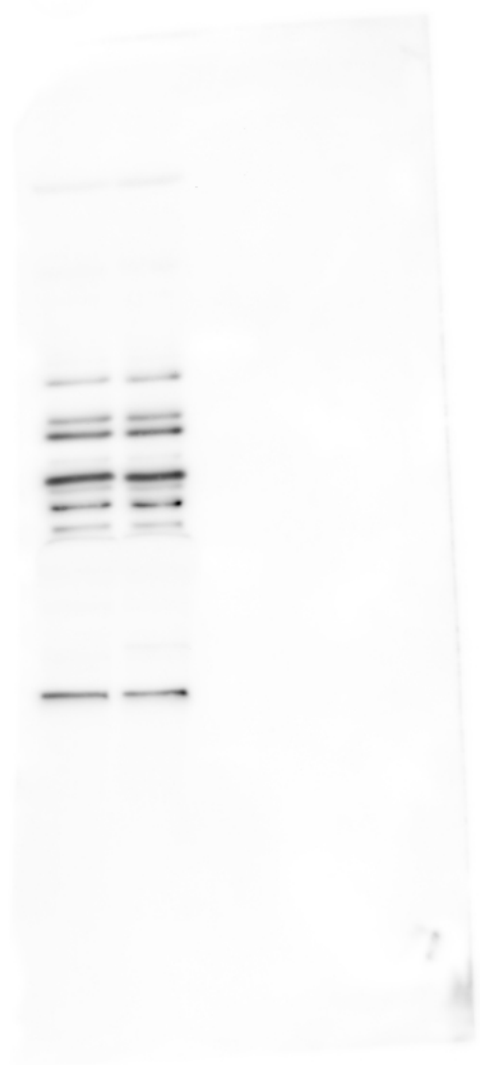

Supplement: Source data 1. [file elife-73511-data1.zip › Source data 1/Figure 2A P2RY14-source data 1.pdf]

|                     | 2-5 min |   |      |     |     |     |
|---------------------|---------|---|------|-----|-----|-----|
| p28 <sup>14</sup> : | -       | - | 62.5 | 125 | 250 | 500 |
| EGF                 | -       | + | +    | +   | +   | +   |

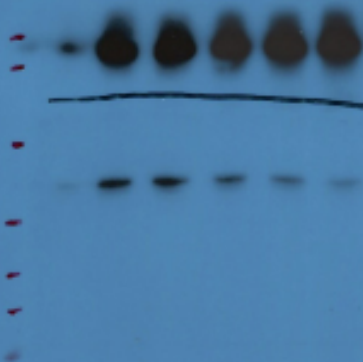

Supplement: Source data 1. [file elife-73511-data1.zip › Source data 1/figure 3E pAKT new-source data 1.pdf]

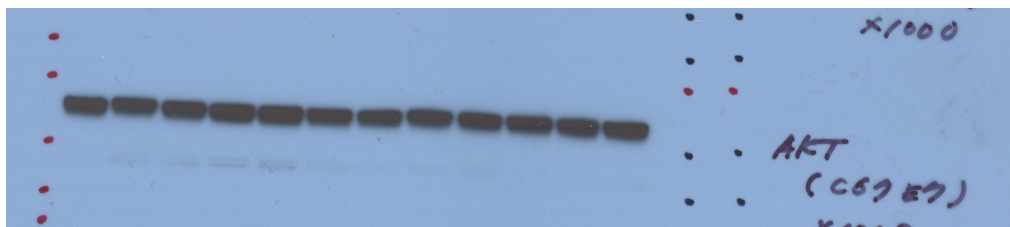

Supplement: Source data 1. [file elife-73511-data1.zip › Source data 1/Figure 3F AKT-source data 1.pdf]

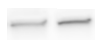

Supplement: Source data 1. [file elife-73511-data1.zip › Source data 1/Figure 2F Nf ERK1-2-source data 1.pdf]

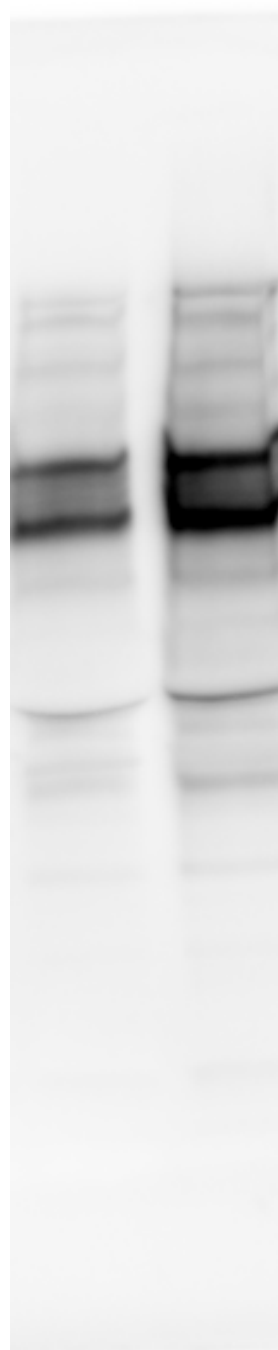

Supplement: Source data 1. [file elife-73511-data1.zip › Source data 1/Figure 2G pPKA WT-source data 1.pdf]

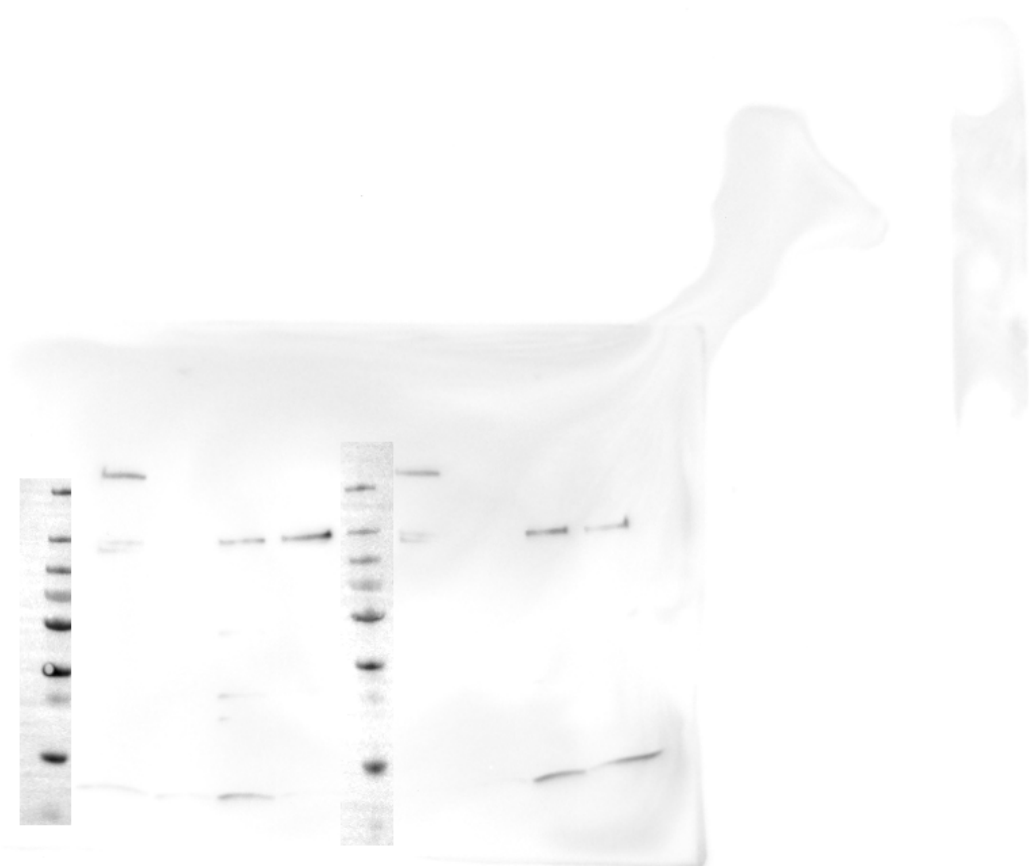

Supplement: Source data 1. [file elife-73511-data1.zip › Source data 1/Figure 3A NF1-source data 1.pdf]

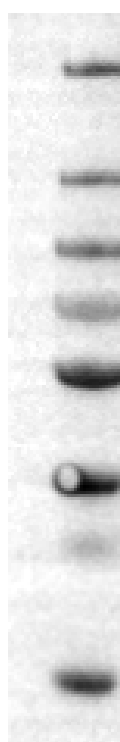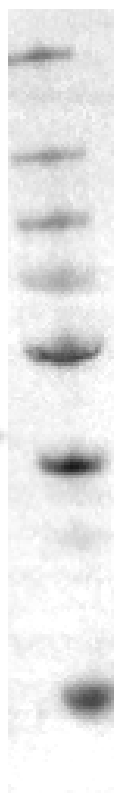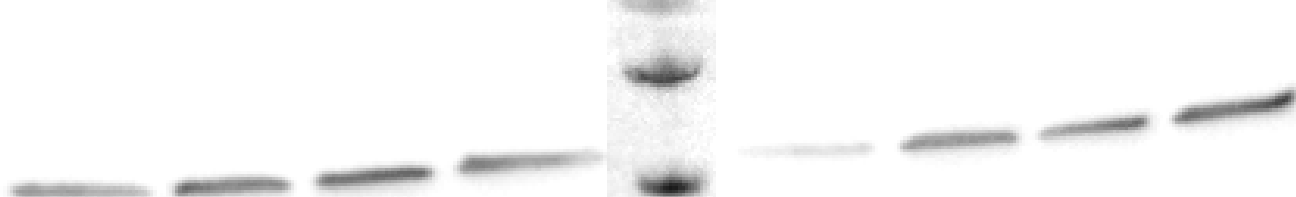

Supplement: Source data 1. [file elife-73511-data1.zip › Source data 1/Figure 3A gapdh-source data 1.pdf]

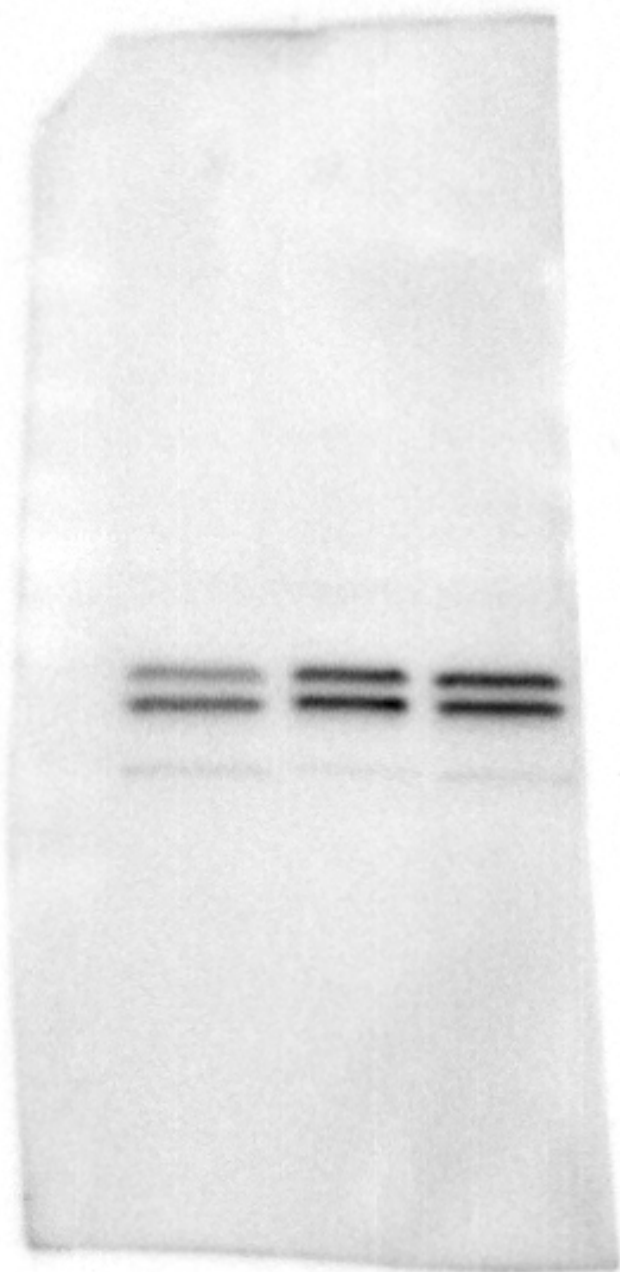

Supplement: Source data 1. [file elife-73511-data1.zip › Source data 1/Figure 5I pERK1-2-source data 1.pdf]

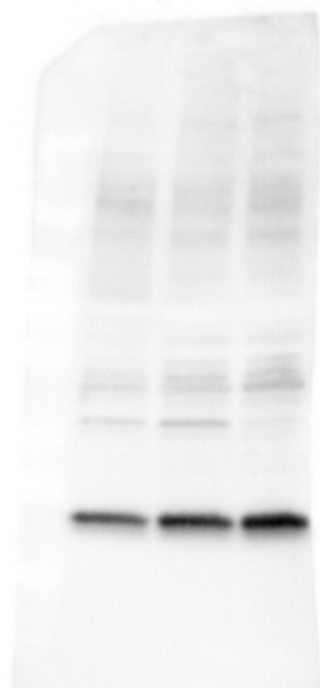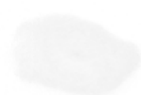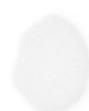

Supplement: Source data 1. [file elife-73511-data1.zip › Source data 1/Figure 4C sciatic P2RY14-source data 1.pdf]

20 min.

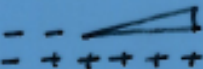

-

-

-

-

- 50

- 37

-

-

Supplement: Source data 1. [file elife-73511-data1.zip › Source data 1/Figure 3E b-actin-source data 1.pdf]

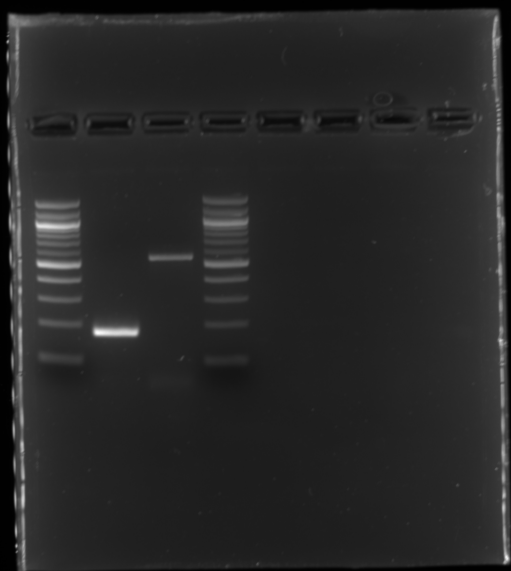

Supplement: Source data 1. [file elife-73511-data1.zip › Source data 1/Figure 4B-source data 1.pdf]

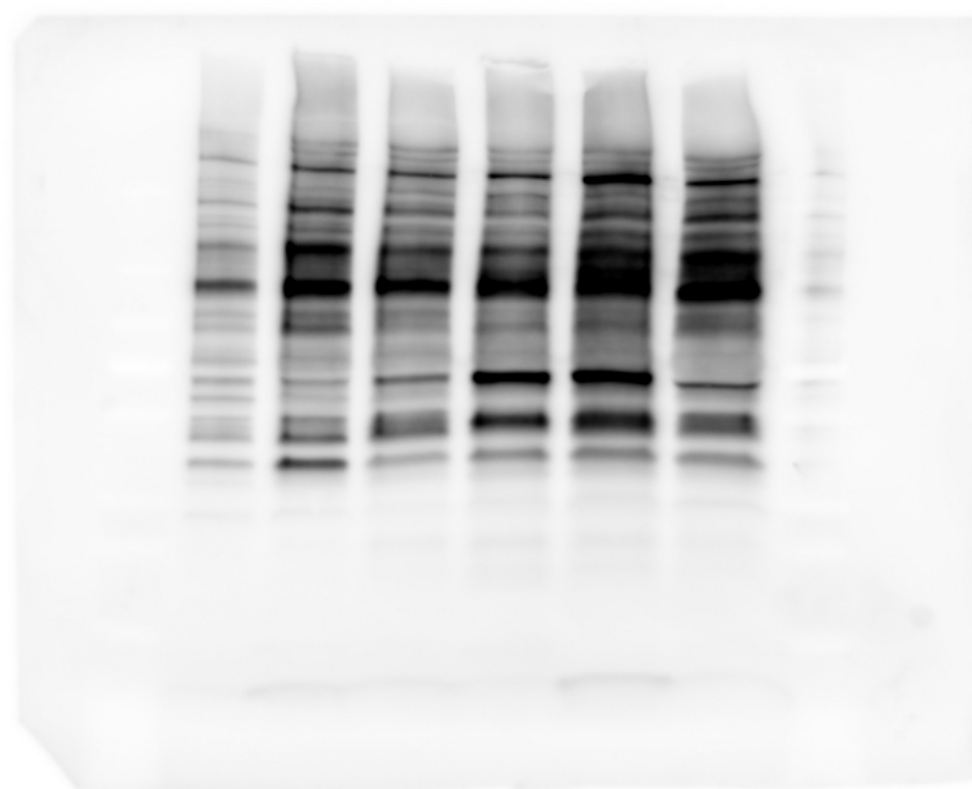

Supplement: Source data 1. [file elife-73511-data1.zip › Source data 1/Figure 6B-pPKA-source data 1.pdf]

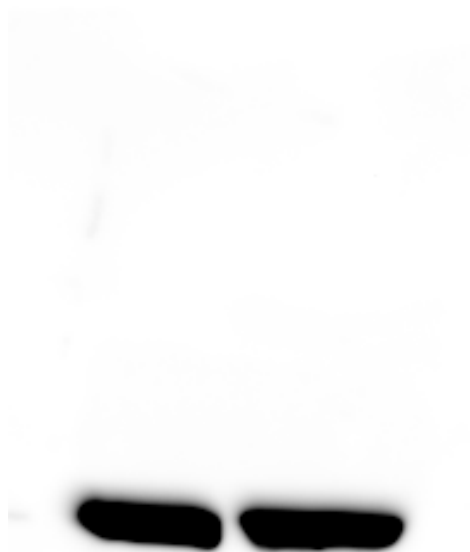

Supplement: Source data 1. [file elife-73511-data1.zip › Source data 1/Figure S3E b-actin-source data 1.pdf]

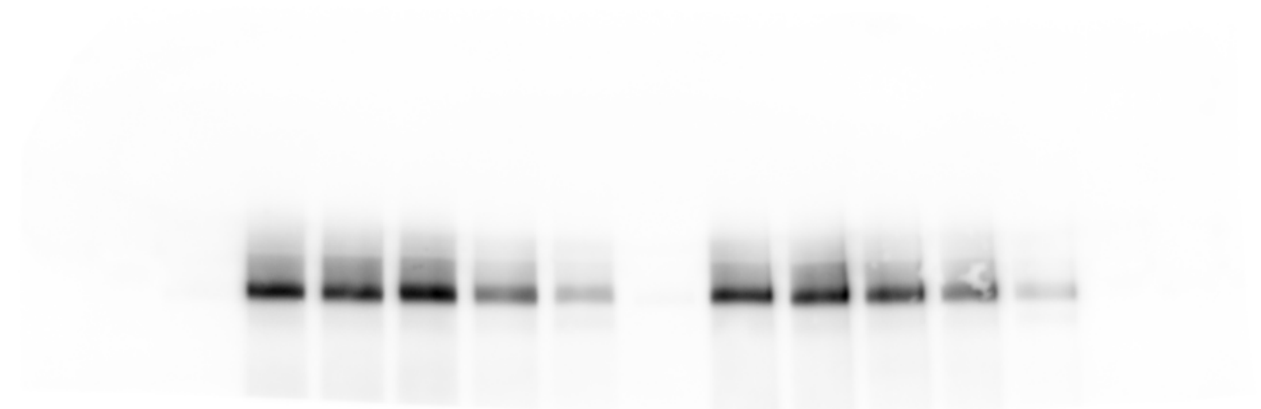

Supplement: Source data 1. [file elife-73511-data1.zip › Source data 1/Figure 3D 1 lambda p-EGFR-source data 1.pdf]

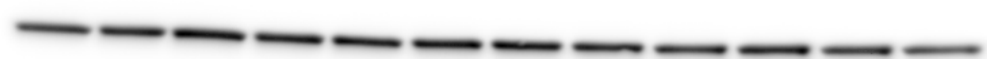

Supplement: Source data 1. [file elife-73511-data1.zip › Source data 1/Figure 3D 1 lambda b-actin-source data 1.pdf]

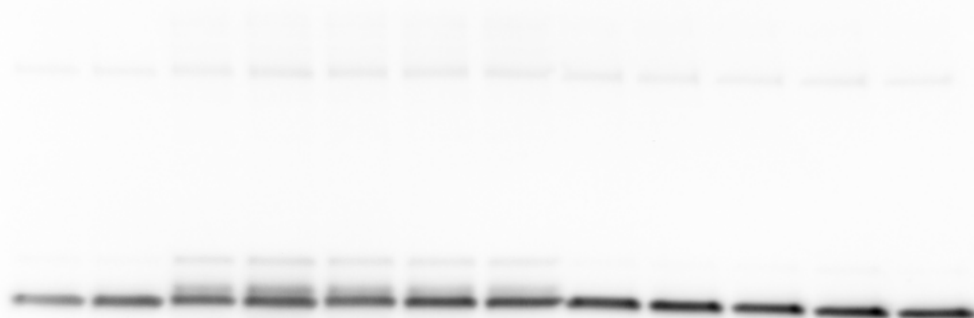

Supplement: Source data 1. [file elife-73511-data1.zip › Source data 1/Figure 3C delta NF pCREB-source data 1.pdf]

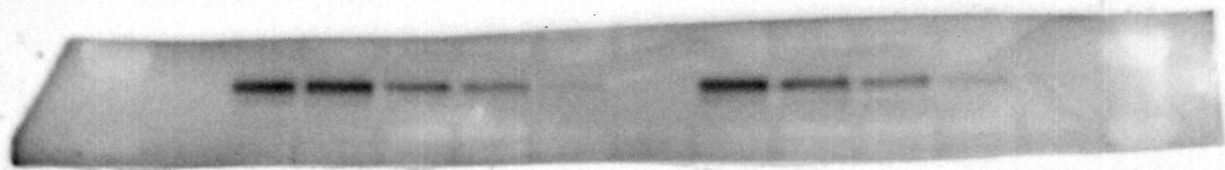

Supplement: Source data 1. [file elife-73511-data1.zip › Source data 1/Figure 3D 1 lambda pAKT-source data 1.pdf]

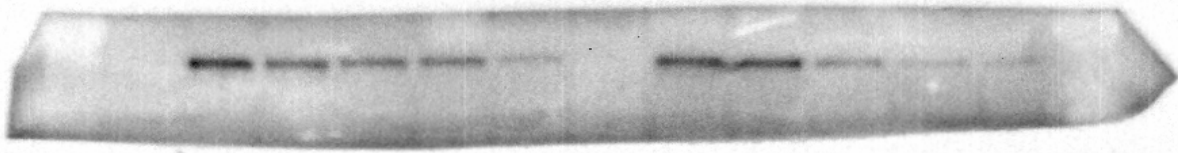

Supplement: Source data 1. [file elife-73511-data1.zip › Source data 1/Figure 3D delta nf pAKT-source data 1.pdf]

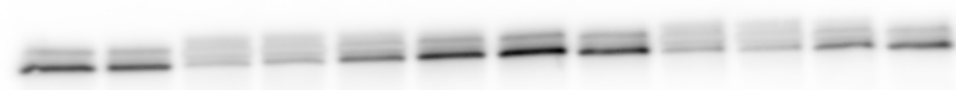

Supplement: Source data 1. [file elife-73511-data1.zip › Source data 1/Figure 3D 1 lambda total ERK1-2-source data 1.pdf]

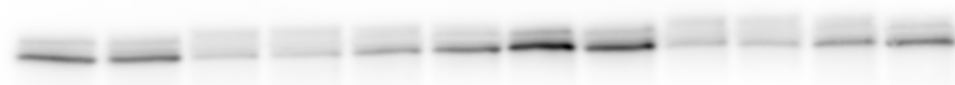

Supplement: Source data 1. [file elife-73511-data1.zip › Source data 1/Figure 3D delta nf total ERK1-2-source data 1.pdf]

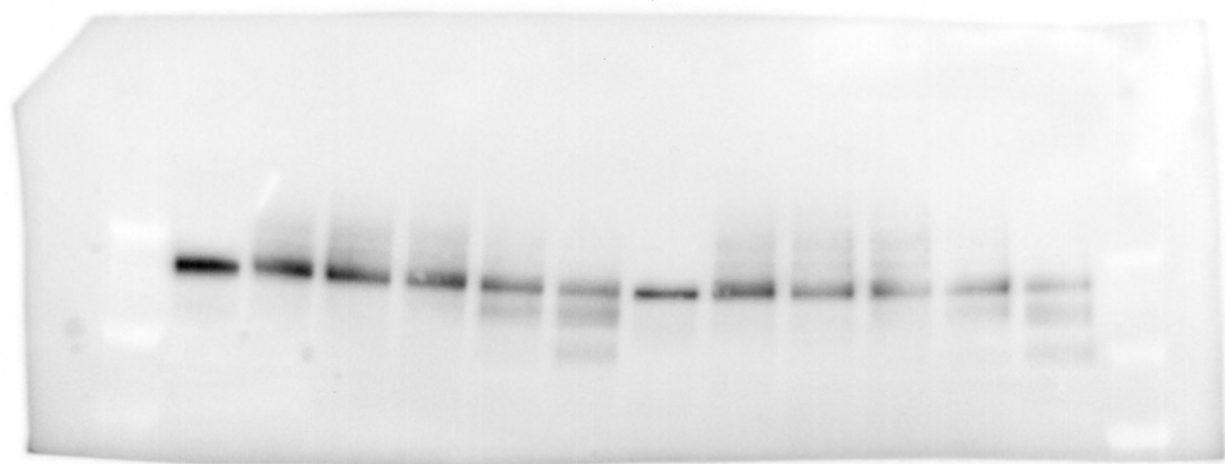

Supplement: Source data 1. [file elife-73511-data1.zip › Source data 1/Figure 3D 1 lambda total EGFR-source data 1.pdf]

p2Y141

EGF

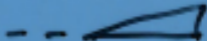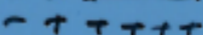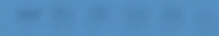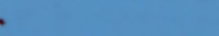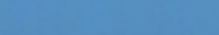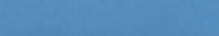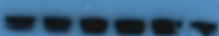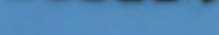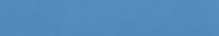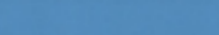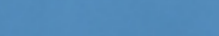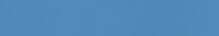

Supplement: Source data 1. [file elife-73511-data1.zip › Source data 1/Figure 3E ERK1-2-source data 1.pdf]

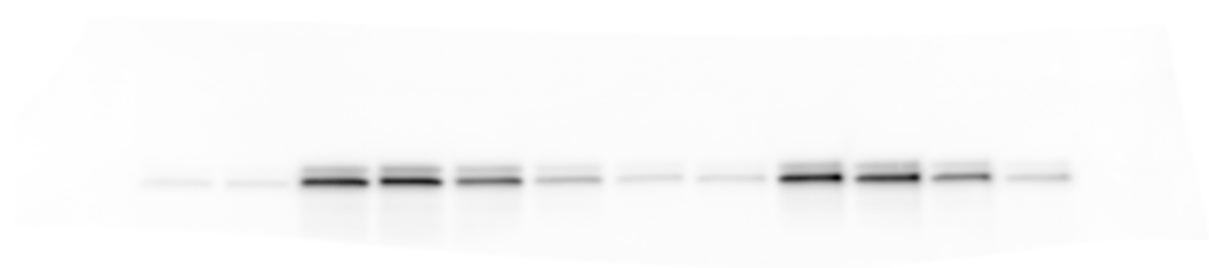

Supplement: Source data 1. [file elife-73511-data1.zip › Source data 1/Figure 3D 1 lambda Perk1-2-source data 1.pdf]

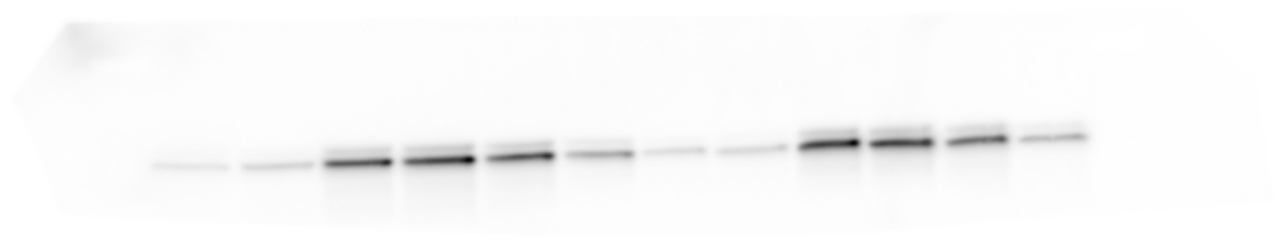

Supplement: Source data 1. [file elife-73511-data1.zip › Source data 1/Figure 3D delta NF pERK1-2-source data 1.pdf]

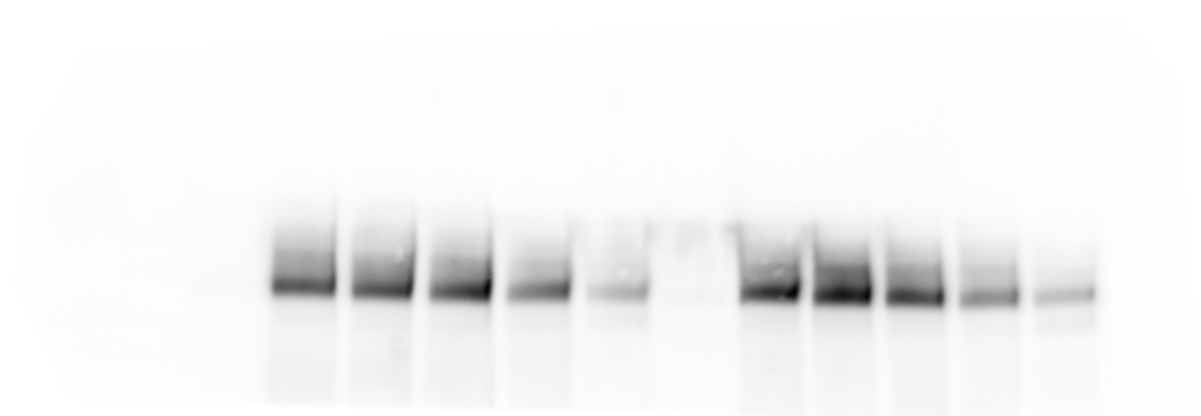

Supplement: Source data 1. [file elife-73511-data1.zip › Source data 1/Figure 3D delta NF p-EGFR-source data 1.pdf]

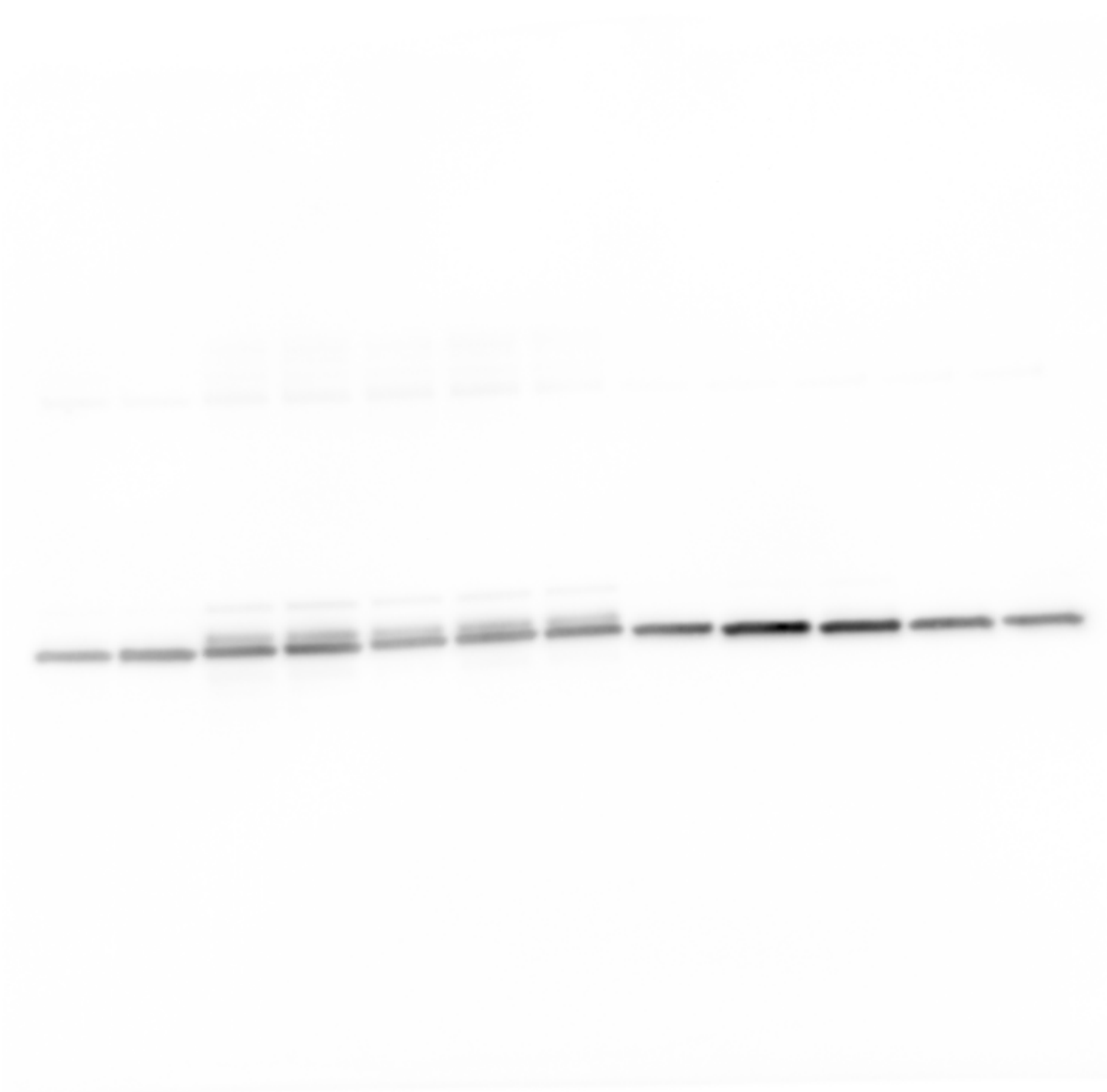

Supplement: Source data 1. [file elife-73511-data1.zip › Source data 1/Figure 3C 1 lambda pCREB-source data 1.pdf]

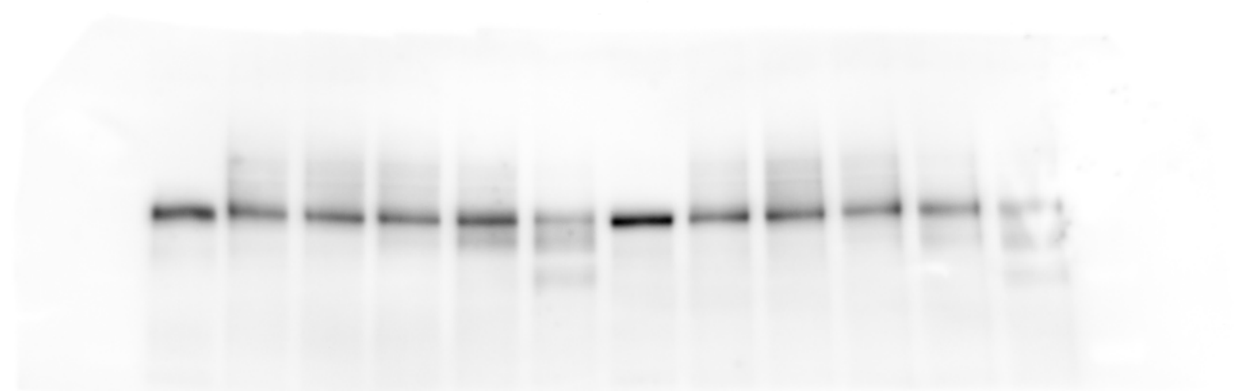

Supplement: Source data 1. [file elife-73511-data1.zip › Source data 1/Figure 3D delta nf total EGFR-source data 1.pdf]

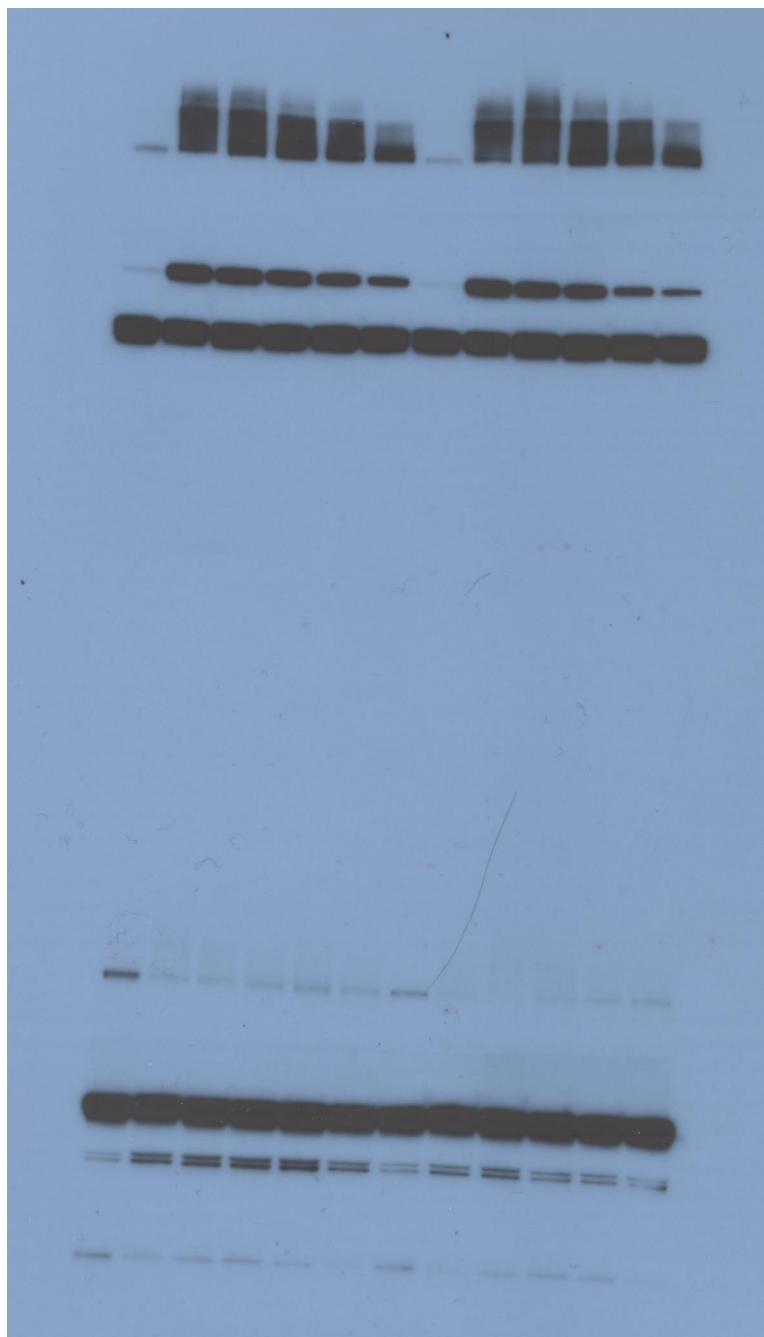

Supplement: Source data 1. [file elife-73511-data1.zip › Source data 1/Figure 3F pERK and pEGFR-source data 1.pdf]

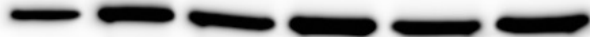

Supplement: Source data 1. [file elife-73511-data1.zip › Source data 1/Figure 6B bactin-source data 1.pdf]

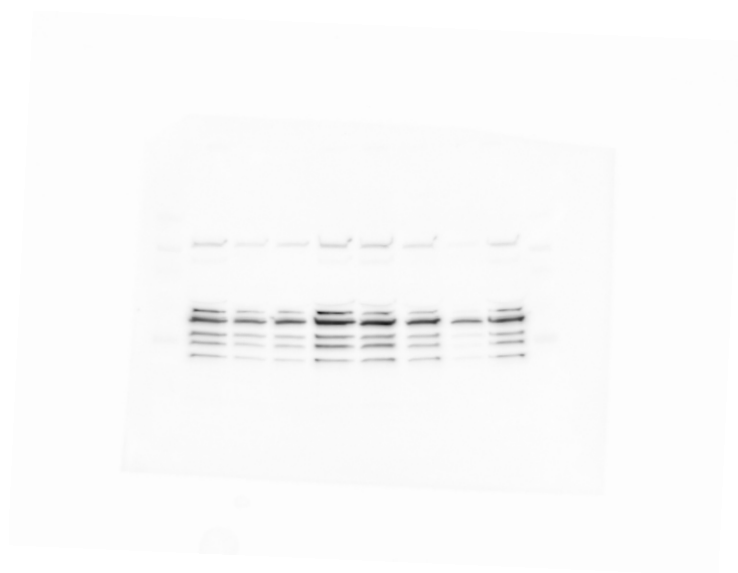

Supplement: Source data 1. [file elife-73511-data1.zip › Source data 1/Figure 2D P2RY14-source data 1.pdf]

8/27/08  
Actin

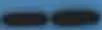

Supplement: Source data 1. [file elife-73511-data1.zip › Source data 1/figure 1B b-actin-source data 1.pdf]

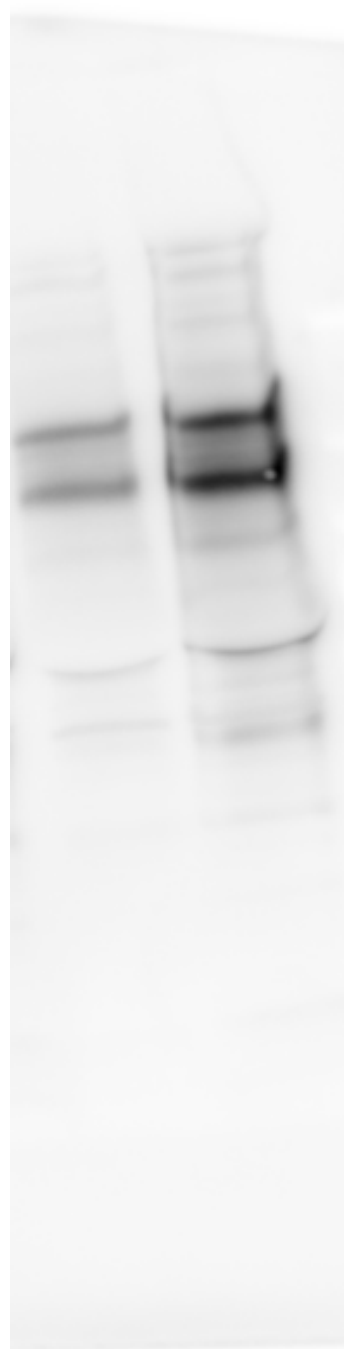

Supplement: Source data 1. [file elife-73511-data1.zip › Source data 1/Figure 2G pPKA Nf-source data 1.pdf]

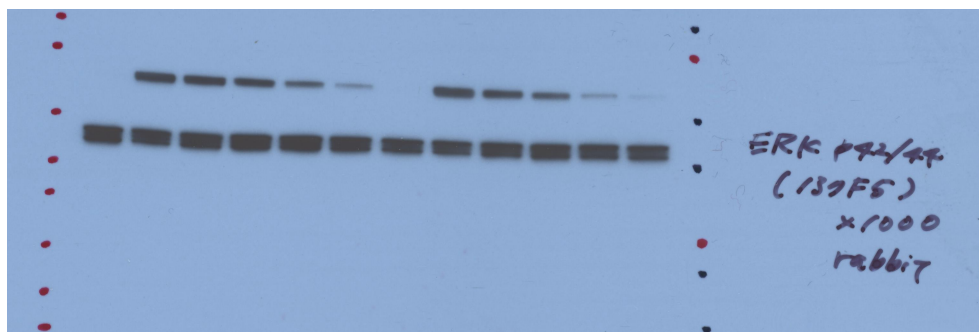

Supplement: Source data 1. [file elife-73511-data1.zip › Source data 1/Figure 3F total ERK and pAKT-source data 1.pdf]

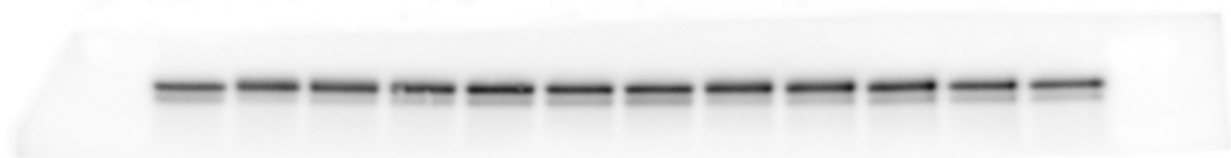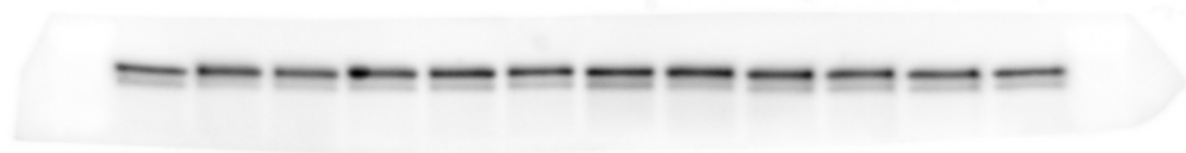

Supplement: Source data 1. [file elife-73511-data1.zip › Source data 1/Figure 3D 1 lambda top and delta NF bottom total AKT-source data 1.pdf]

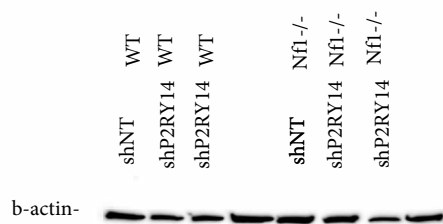

Supplement: Source data 2. [file elife-73511-data2.zip › Source data 2/Figure 2D b-actin-source data 2.pdf]

# ST88-14 MPNST cells

p214i - - 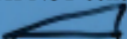  
 EGF - + + + + +

ERK1/2-

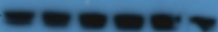

Supplement: Source data 2. [file elife-73511-data2.zip › Source data 2/Figure 3E ERK1-2-source data 2.pdf]

# ST88-14 MPNST cells

P2RY14i - - - - - + + + + +

EGF 0 5 10 15 20 30 0 5 10 15 20 30 EGF (min)

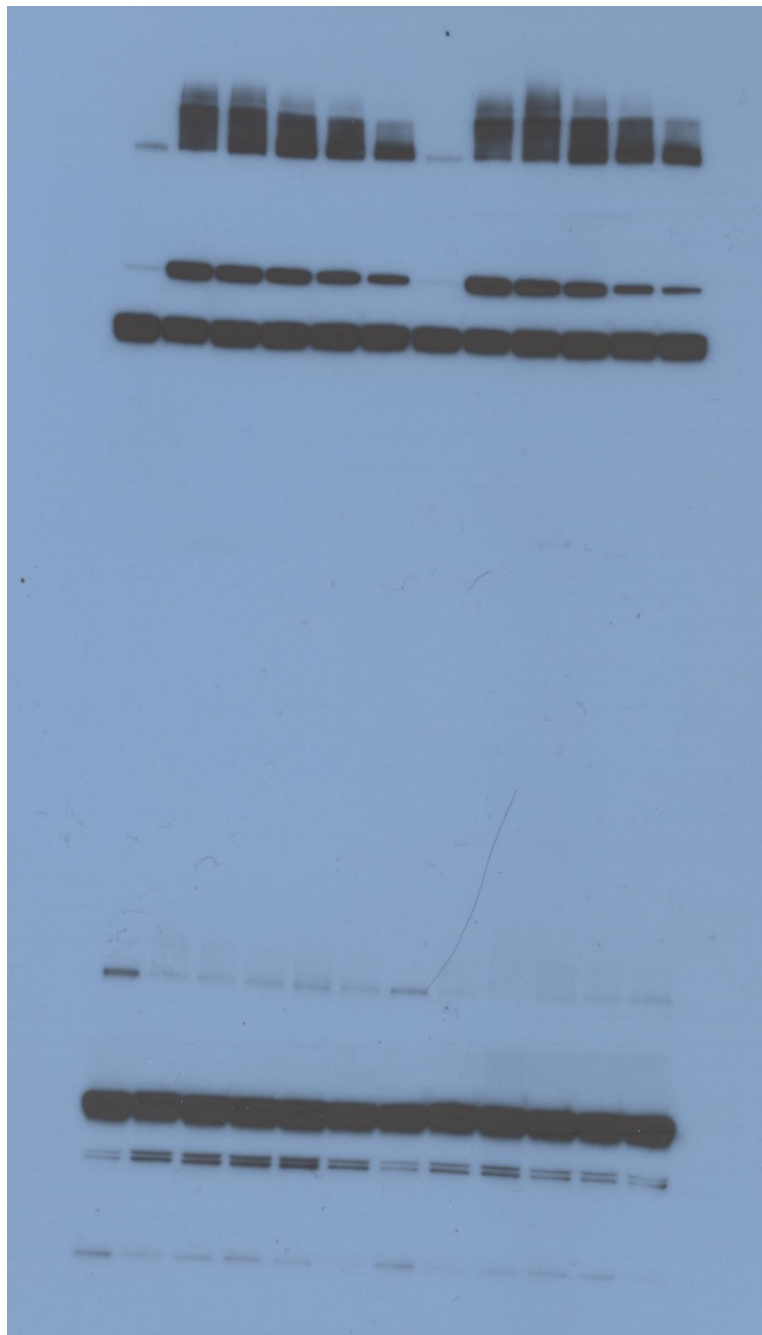

- pEGFR  
(Y1068;Grb2)

- pERK1/2  
(T202)/Y204

Supplement: Source data 2. [file elife-73511-data2.zip › Source data 2/Figure 3F pERK and pEGFR-source data 2.pdf]

ST88-14 MPNST cells

|         |   |   |    |    |    |    |   |   |    |    |    |    |
|---------|---|---|----|----|----|----|---|---|----|----|----|----|
| P2RY14i | - | - | -  | -  | -  | -  | + | + | +  | +  | +  | +  |
| EGF     | 0 | 5 | 10 | 15 | 20 | 30 | 0 | 5 | 10 | 15 | 20 | 30 |

EGF (min)

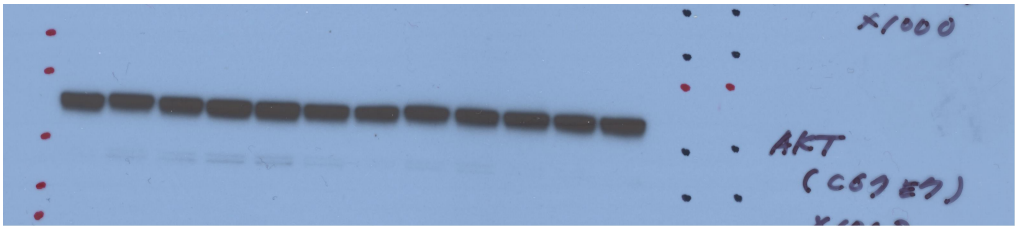

Supplement: Source data 2. [file elife-73511-data2.zip › Source data 2/Figure 3F AKT-source data 1.pdf]

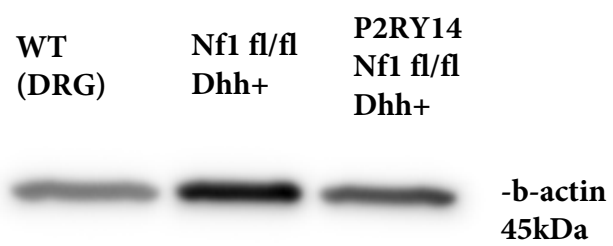

Supplement: Source data 2. [file elife-73511-data2.zip › Source data 2/Figure 5I b-actin-source data 1.pdf]

WT      Nf1<sup>-/-</sup>

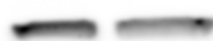 - P2RY14

Supplement: Source data 2. [file elife-73511-data2.zip › Source data 2/Figure S3E P2RY14-source data 2.pdf]

Nf1<sup>-/-</sup>

shNT  
shP2RY14

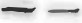

-pERK1/2

Supplement: Source data 2. [file elife-73511-data2.zip › Source data 2/Figure 2F NF pERK1-2-source data 2.pdf]

Nf1<sup>-/-</sup>

shNT  
shP2RY14

-ERK1/2

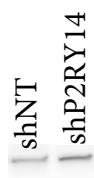

Supplement: Source data 2. [file elife-73511-data2.zip › Source data 2/Figure 2F Nf ERK1-2-source data 2.pdf]

WT

shNT shP2RY14

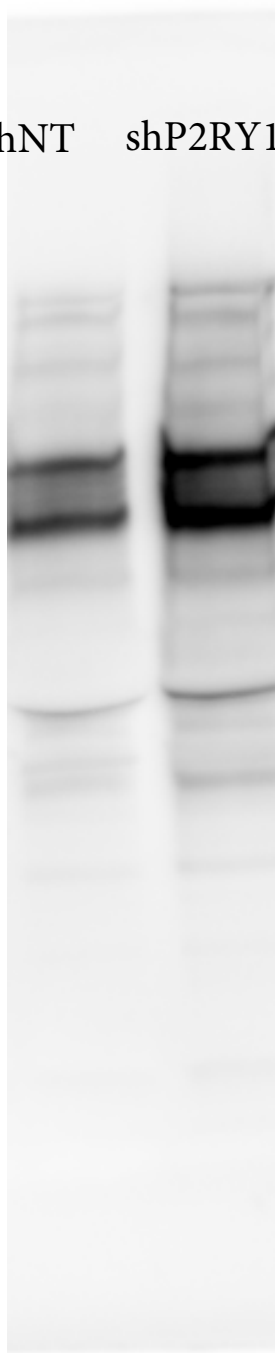

Supplement: Source data 2. [file elife-73511-data2.zip › Source data 2/Figure 2G pPKA WT-source data 2.pdf]

Nf1<sup>-/-</sup>

shNT    shP2RY14

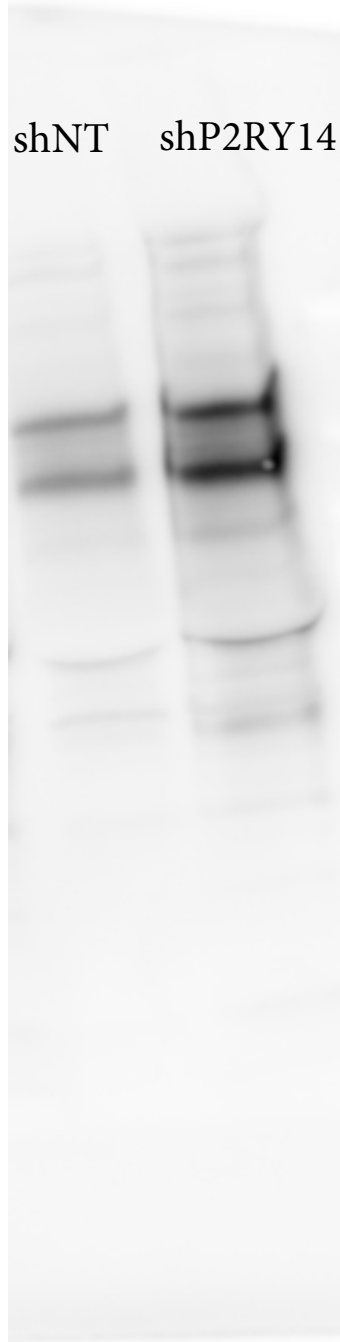

Supplement: Source data 2. [file elife-73511-data2.zip › Source data 2/Figure 2G pPKA Nf-source data 2.pdf]

ST88-14 MPNST cells

57

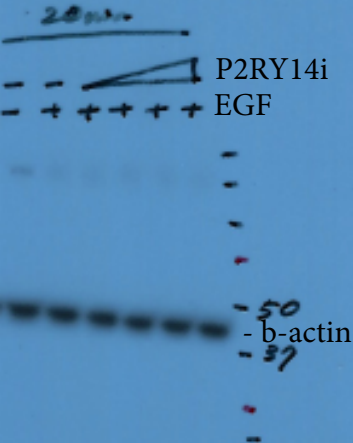

Supplement: Source data 2. [file elife-73511-data2.zip › Source data 2/Figure 3E b-actin-source data 1.pdf]

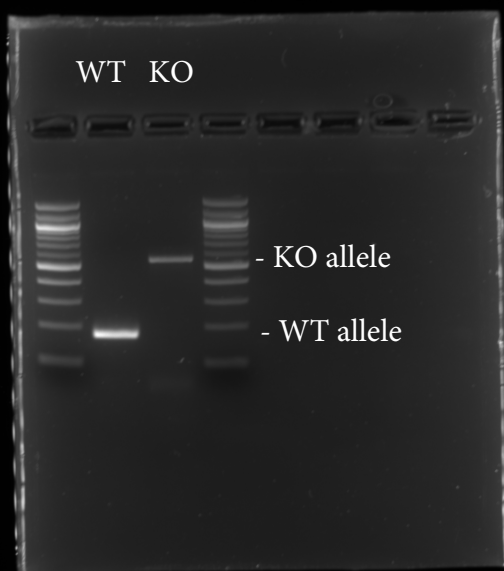

Supplement: Source data 2. [file elife-73511-data2.zip › Source data 2/Figure 4B-source data 1.pdf]

**WT  
(DRG)**

**Nf1 fl/fl  
Dhh+**

**P2RY14  
Nf1 fl/fl  
Dhh+**

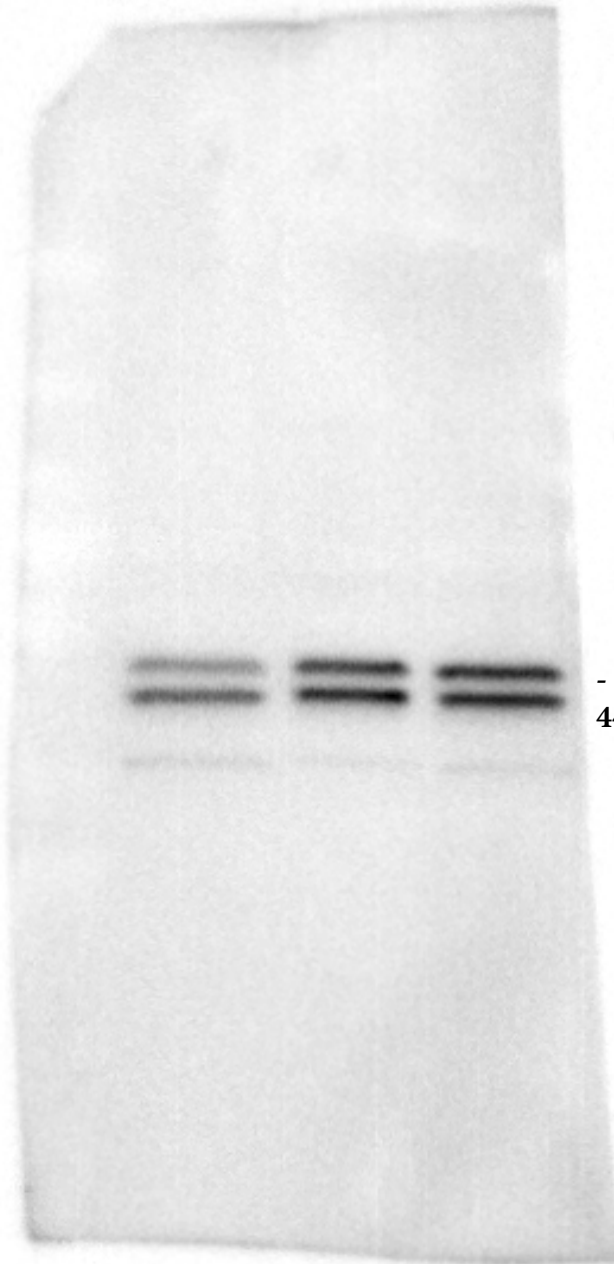

**- p-ERK 1/2  
44/42 kDa**

Supplement: Source data 2. [file elife-73511-data2.zip › Source data 2/Figure 5I pERK1-2-source data 2.pdf]

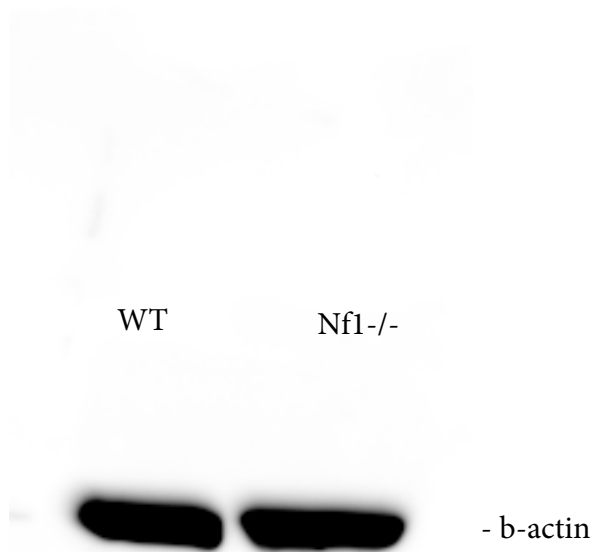

Supplement: Source data 2. [file elife-73511-data2.zip › Source data 2/Figure S3E b-actin-source data 1.pdf]

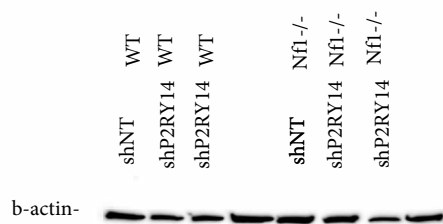

Supplement: Source data 2. [file elife-73511-data2.zip › Source data 2/Figure 2F b-actin-source data 2.pdf]

1 lambda

|                  |   |   |   |   |   |   |   |   |   |   |   |   |
|------------------|---|---|---|---|---|---|---|---|---|---|---|---|
| UDP              | - | - | - | - | - | + | + | + | - | + | + | + |
| P2RY14 inhibitor | - | - | - | - | + | - | + | + | + | - | - | - |
| IBMX             | + | - | + | - | + | + | + | + | + | + | + | + |

p-CREB-

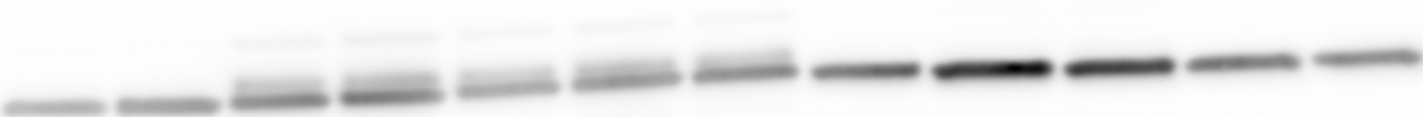

Supplement: Source data 2. [file elife-73511-data2.zip › Source data 2/Figure 3C 1 lambda pCREB-source data 2.pdf]

1 lambda  
delta NF

|                     |   |   |   |   |   |   |   |   |   |   |   |   |
|---------------------|---|---|---|---|---|---|---|---|---|---|---|---|
| UDP                 | - | - | - | - | - | + | + | + | - | + | + | + |
| P2RY14<br>inhibitor | - | - | - | - | + | - | + | + | + | - | - | - |
| IBMX                | + | - | + | - | + | + | + | + | + | + | + | + |

p-CREB-

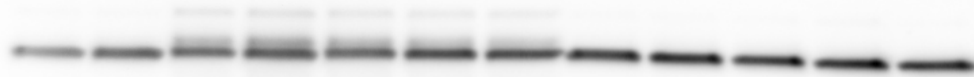

Supplement: Source data 2. [file elife-73511-data2.zip › Source data 2/Figure 3C delta NF pCREB-source data 2.pdf]

**1 lambda**

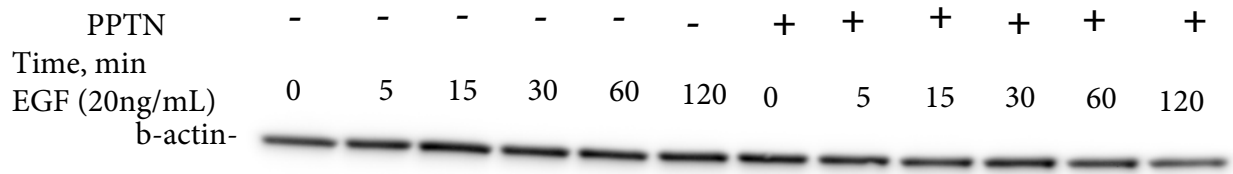

Supplement: Source data 2. [file elife-73511-data2.zip › Source data 2/Figure 3D 1 lambda b-actin-source data 2.pdf]

1 lambda

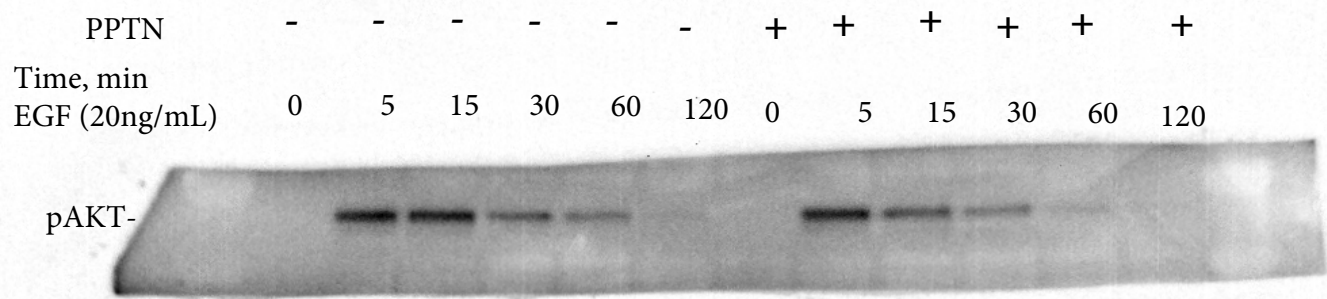

Supplement: Source data 2. [file elife-73511-data2.zip › Source data 2/Figure 3D 1 lambda pAKT-source data 2.pdf]

**1 lambda delta Nf1**

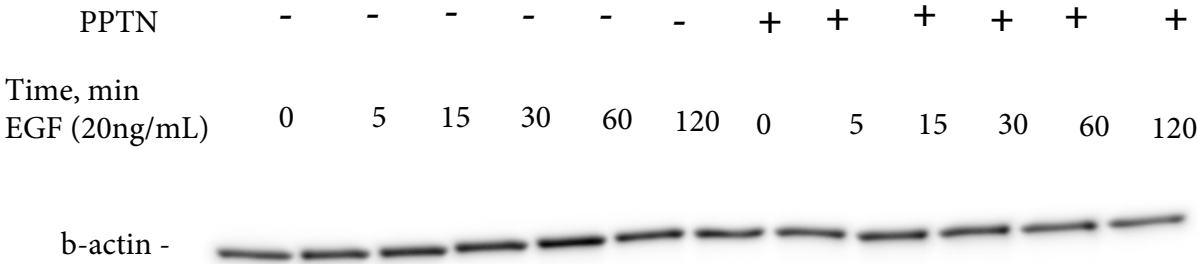

Supplement: Source data 2. [file elife-73511-data2.zip › Source data 2/Figure 3D delta NF b-actin-source data 2.pdf]

# 1 lambda

|               |   |   |    |    |    |     |   |   |    |    |    |     |
|---------------|---|---|----|----|----|-----|---|---|----|----|----|-----|
| PPTN          | - | - | -  | -  | -  | -   | + | + | +  | +  | +  | +   |
| Time, min     | 0 | 5 | 15 | 30 | 60 | 120 | 0 | 5 | 15 | 30 | 60 | 120 |
| EGF (20ng/mL) |   |   |    |    |    |     |   |   |    |    |    |     |

total-ERK1/2 -

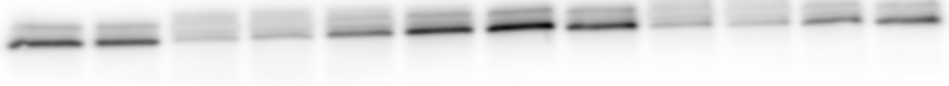

Supplement: Source data 2. [file elife-73511-data2.zip › Source data 2/Figure 3D 1 lambda total ERK1-2-source data 2.pdf]

# ST88-14 MPNST cells

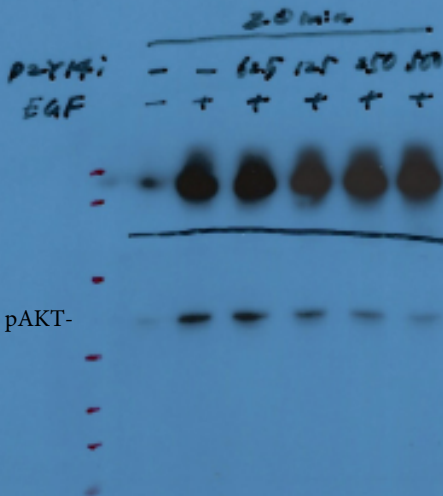

Supplement: Source data 2. [file elife-73511-data2.zip › Source data 2/figure 3E pAKT new-source data 3.pdf]

# 1 lambda delta Nf1

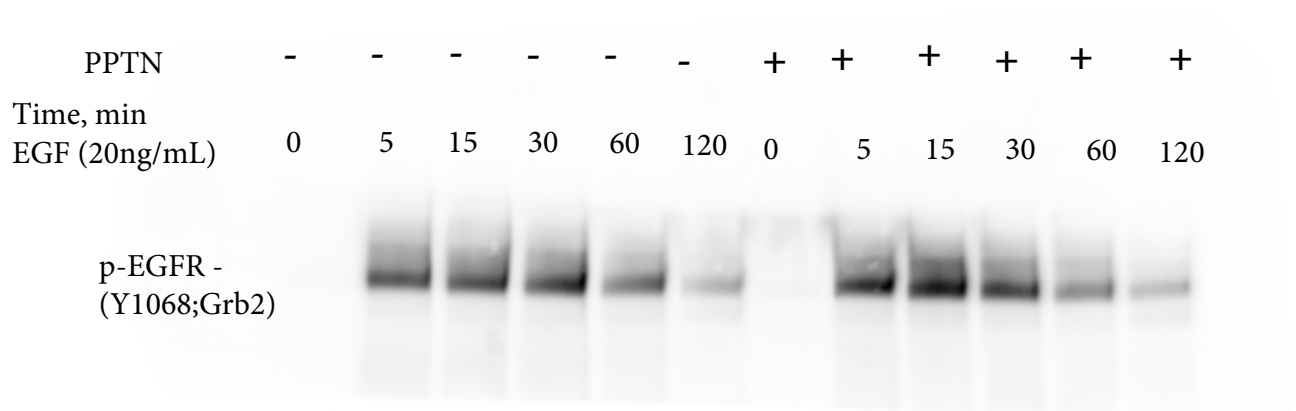

Supplement: Source data 2. [file elife-73511-data2.zip › Source data 2/Figure 3D delta NF p-EGFR-source data 2.pdf]

# 1 lambda

|               |   |   |    |    |    |     |   |   |    |    |    |     |
|---------------|---|---|----|----|----|-----|---|---|----|----|----|-----|
| PPTN          | - | - | -  | -  | -  | -   | + | + | +  | +  | +  | +   |
| Time, min     | 0 | 5 | 15 | 30 | 60 | 120 | 0 | 5 | 15 | 30 | 60 | 120 |
| EGF (20ng/mL) |   |   |    |    |    |     |   |   |    |    |    |     |

total-EGFR-

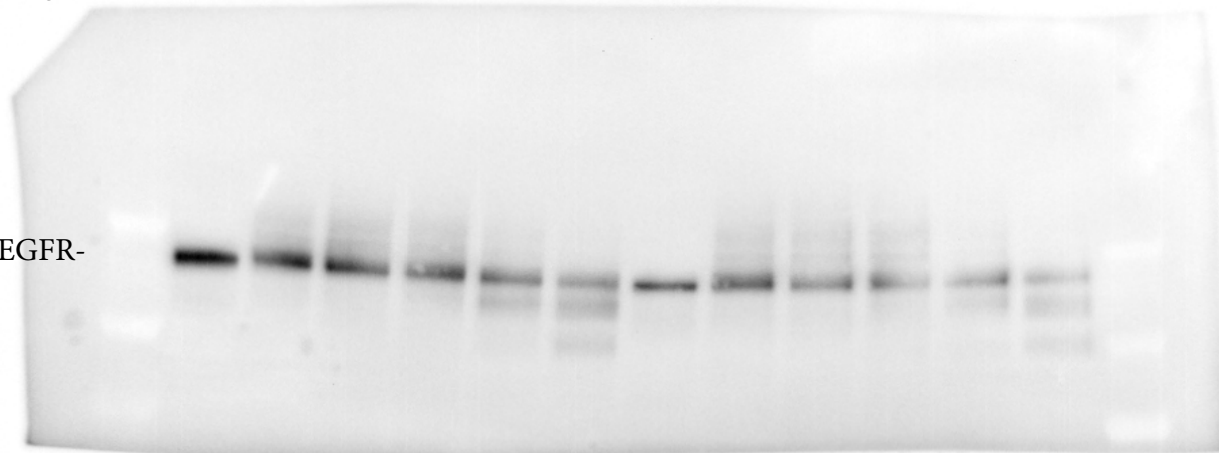

Supplement: Source data 2. [file elife-73511-data2.zip › Source data 2/Figure 3D 1 lambda total EGFR-source data 2.pdf]

1 lambda

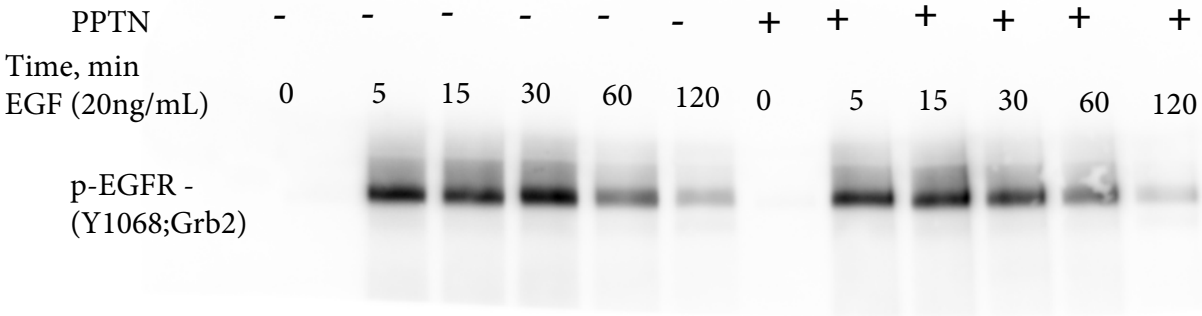

Supplement: Source data 2. [file elife-73511-data2.zip › Source data 2/Figure 3D 1 lambda p-EGFR-source data 2.pdf]

1 lambda delta Nf1

|               |   |   |    |    |    |     |   |   |    |    |    |     |
|---------------|---|---|----|----|----|-----|---|---|----|----|----|-----|
| PPTN          | - | - | -  | -  | -  | -   | + | + | +  | +  | +  | +   |
| Time, min     | 0 | 5 | 15 | 30 | 60 | 120 | 0 | 5 | 15 | 30 | 60 | 120 |
| EGF (20ng/mL) |   |   |    |    |    |     |   |   |    |    |    |     |

p-AKT -

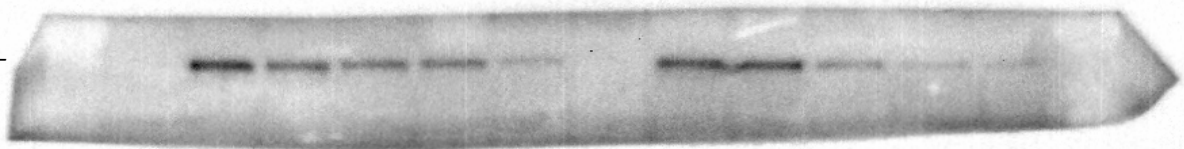

Supplement: Source data 2. [file elife-73511-data2.zip › Source data 2/Figure 3D delta nf pAKT-source data 2.pdf]

# 1 lambda delta Nf1

|               |   |   |    |    |    |     |   |   |    |    |    |     |
|---------------|---|---|----|----|----|-----|---|---|----|----|----|-----|
| PPTN          | - | - | -  | -  | -  | -   | + | + | +  | +  | +  | +   |
| Time, min     | 0 | 5 | 15 | 30 | 60 | 120 | 0 | 5 | 15 | 30 | 60 | 120 |
| EGF (20ng/mL) |   |   |    |    |    |     |   |   |    |    |    |     |

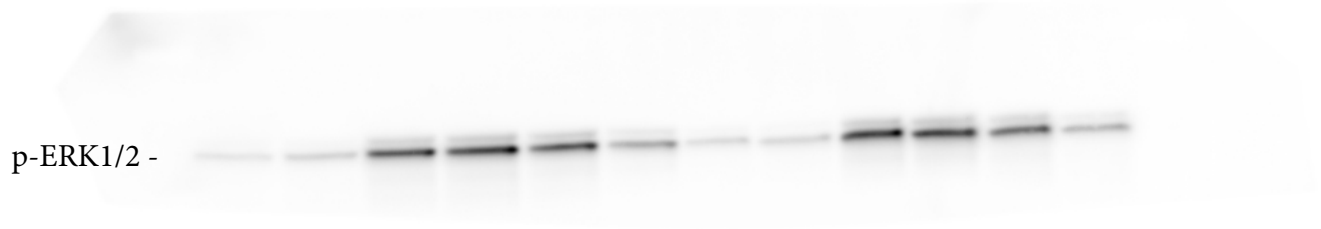

Supplement: Source data 2. [file elife-73511-data2.zip › Source data 2/Figure 3D delta NF pERK1-2-source data 2.pdf]

1 lambda delta Nf1

|               |   |   |    |    |    |     |   |   |    |    |    |     |
|---------------|---|---|----|----|----|-----|---|---|----|----|----|-----|
| PPTN          | - | - | -  | -  | -  | -   | + | + | +  | +  | +  | +   |
| Time, min     | 0 | 5 | 15 | 30 | 60 | 120 | 0 | 5 | 15 | 30 | 60 | 120 |
| EGF (20ng/mL) |   |   |    |    |    |     |   |   |    |    |    |     |

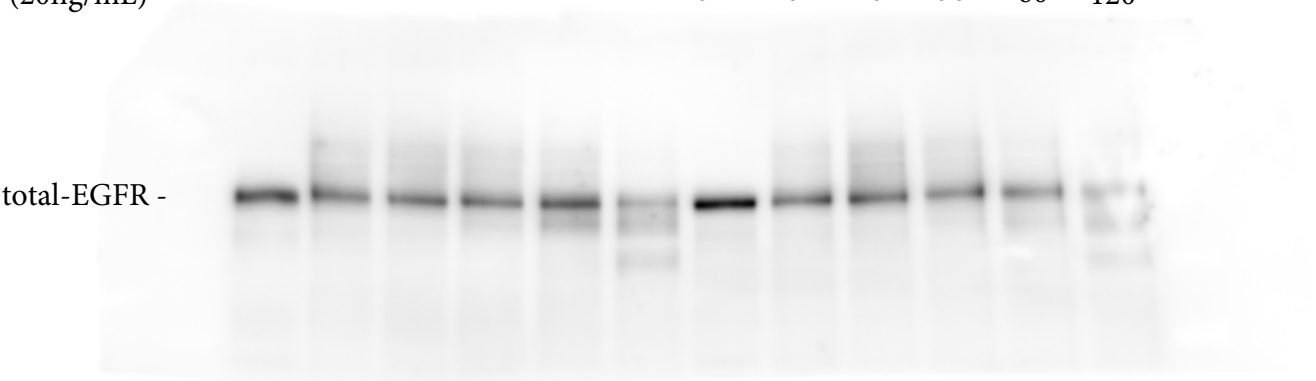

Supplement: Source data 2. [file elife-73511-data2.zip › Source data 2/Figure 3D delta nf total EGFR-source data 2.pdf]

**1 lambda delta Nf1**

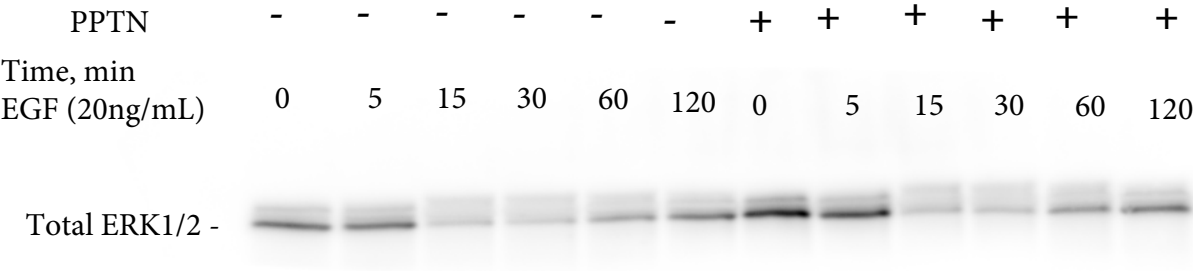

Supplement: Source data 2. [file elife-73511-data2.zip › Source data 2/Figure 3D delta nf total ERK1-2-source data 13.pdf]

1/2

ST88-14 MPNST cells

20

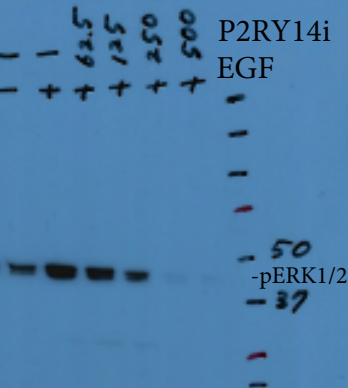

Supplement: Source data 2. [file elife-73511-data2.zip › Source data 2/Figure 3E pERK1-2-source data 4.pdf]

# ST88-14 MPNST cells

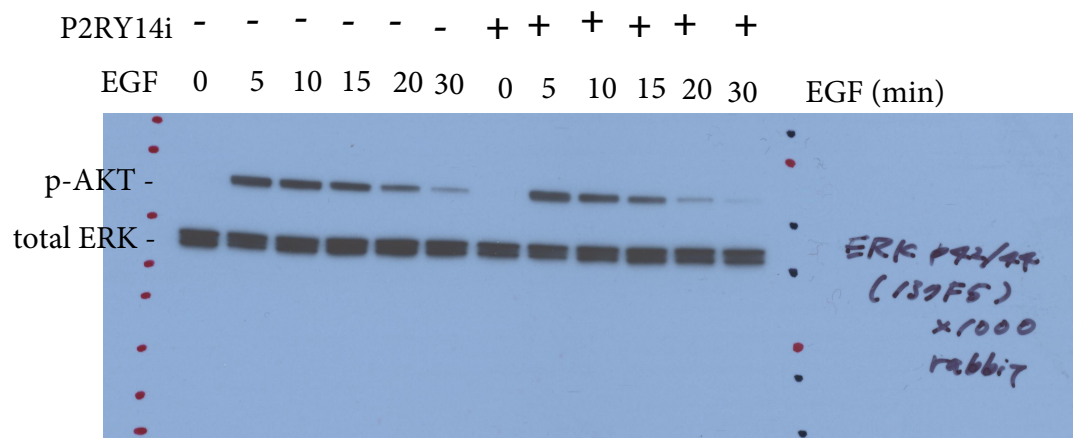

Supplement: Source data 2. [file elife-73511-data2.zip › Source data 2/Figure 3F total ERK and pAKT-source data 3.pdf]

**neurofibroma**

|                  |                  |
|------------------|------------------|
| <b>Nf1 fl/fl</b> | <b>P2RY14</b>    |
| <b>Dhh+</b>      | <b>Nf1 fl/fl</b> |
|                  | <b>Dhh+</b>      |

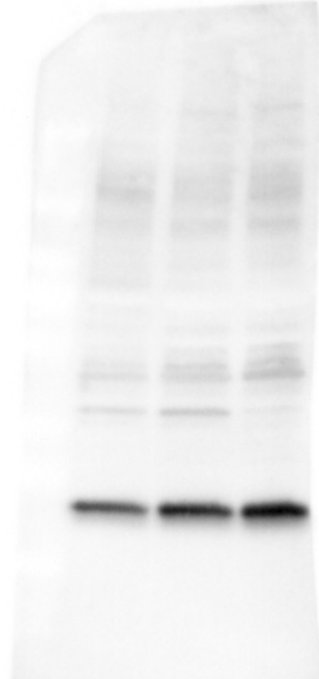

**- P2RY14**

Supplement: Source data 2. [file elife-73511-data2.zip › Source data 2/Figure 4C sciatic P2RY14-source data 4.pdf]

Nf1 fl/fl Dhh+  
Vehicle

---

Nf1 fl/fl Dhh+  
Rolipram 5mg/kg

---

b-actin-  
45 kDa

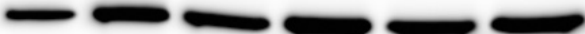

Supplement: Source data 2. [file elife-73511-data2.zip › Source data 2/Figure 6B bactin-source data 1.pdf]

mouse SCP

WT

Nf1<sup>-/-</sup>

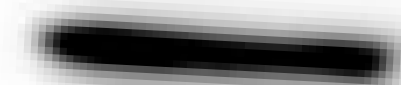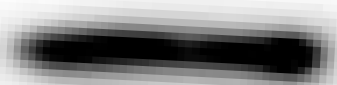

-b-actin

Supplement: Source data 2. [file elife-73511-data2.zip › Source data 2/Figure 2A bactin-source data 2.pdf]

---

mouse SCP

WT Nf1<sup>-/-</sup>

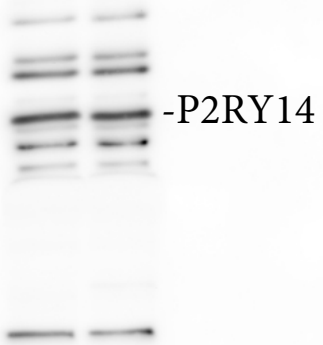

Supplement: Source data 2. [file elife-73511-data2.zip › Source data 2/Figure 2A P2RY14-source data 2.pdf]

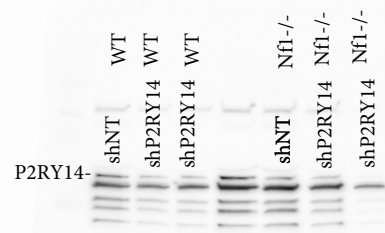

Supplement: Source data 2. [file elife-73511-data2.zip › Source data 2/Figure 2D P2RY14-source data 2.pdf]

shNT  
shP2RY14  
-ERK1/2

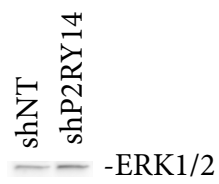

Supplement: Source data 2. [file elife-73511-data2.zip › Source data 2/Figure 2F WT ERK1-2-source data 2.pdf]

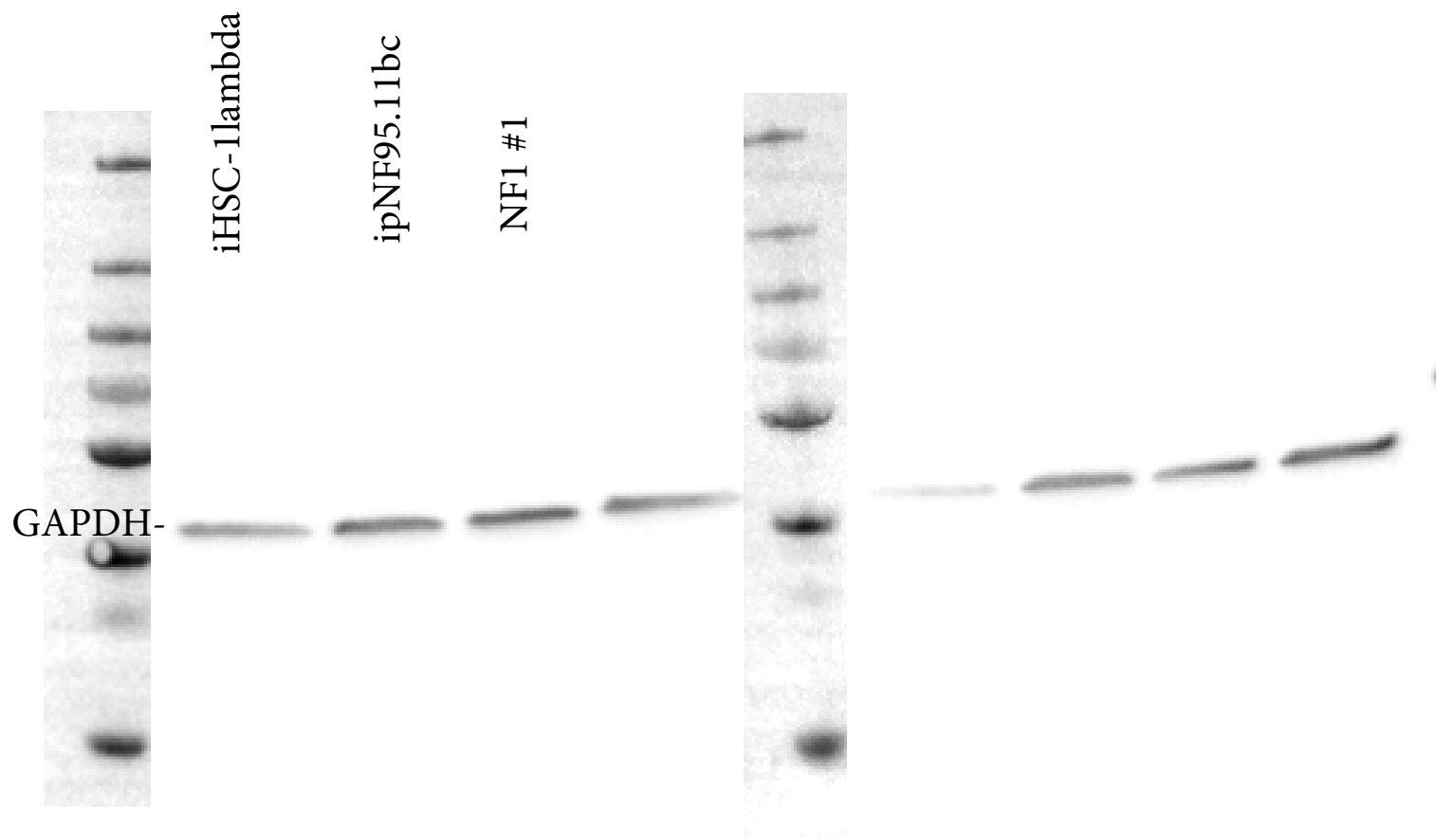

Supplement: Source data 2. [file elife-73511-data2.zip › Source data 2/Figure 3A gapdh-source data 2.pdf]

1 lambda  
delta NF

|                     |                                                                                   |                                                                                   |                                                                                   |                                                                                   |                                                                                   |                                                                                   |                                                                                   |                                                                                   |                                                                                   |                                                                                    |                                                                                     |                                                                                     |
|---------------------|-----------------------------------------------------------------------------------|-----------------------------------------------------------------------------------|-----------------------------------------------------------------------------------|-----------------------------------------------------------------------------------|-----------------------------------------------------------------------------------|-----------------------------------------------------------------------------------|-----------------------------------------------------------------------------------|-----------------------------------------------------------------------------------|-----------------------------------------------------------------------------------|------------------------------------------------------------------------------------|-------------------------------------------------------------------------------------|-------------------------------------------------------------------------------------|
| UDP                 | -                                                                                 | -                                                                                 | -                                                                                 | -                                                                                 | -                                                                                 | +                                                                                 | +                                                                                 | +                                                                                 | -                                                                                 | +                                                                                  | +                                                                                   | +                                                                                   |
| P2RY14<br>inhibitor | -                                                                                 | -                                                                                 | -                                                                                 | -                                                                                 | +                                                                                 | -                                                                                 | +                                                                                 | +                                                                                 | +                                                                                 | -                                                                                  | -                                                                                   | -                                                                                   |
| IBMX                | +                                                                                 | -                                                                                 | +                                                                                 | -                                                                                 | +                                                                                 | +                                                                                 | +                                                                                 | +                                                                                 | +                                                                                 | +                                                                                  | +                                                                                   | +                                                                                   |
| b-actin-            | 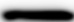 | 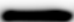 | 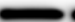 | 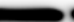 | 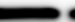 | 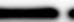 | 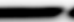 | 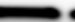 | 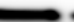 | 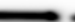 | 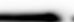 | 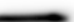 |

Supplement: Source data 2. [file elife-73511-data2.zip › Source data 2/Figure 3C delta NF b-actin-source data 2.pdf]

1 lambda

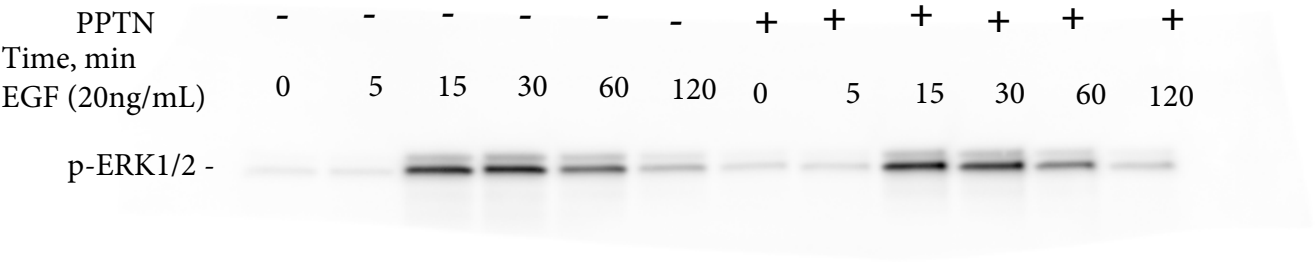

Supplement: Source data 2. [file elife-73511-data2.zip › Source data 2/Figure 3D 1 lambda Perk1-2-source data 2.pdf]

**neurofibroma**

**Nf1 fl/fl  
Dhh+**

**P2RY14  
Nf1 fl/fl  
Dhh+**

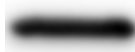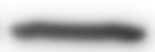

**-b-actin**

Supplement: Source data 2. [file elife-73511-data2.zip › Source data 2/Figure 4C neurofibroma b-actin-source data 1.pdf]

**neurofibroma**

**Nf1 fl/fl  
Dhh+**

**P2RY14  
Nf1 fl/fl  
Dhh+**

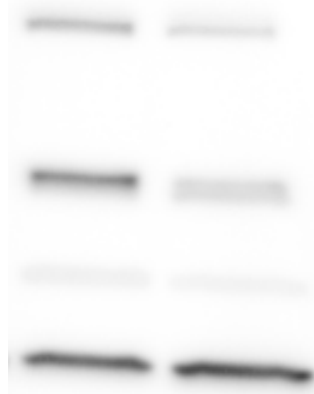

**- P2RY14**

Supplement: Source data 2. [file elife-73511-data2.zip › Source data 2/Figure 4C neurofibroma P2RY14-source data 2.pdf]

**sciatic**

**Nf1 fl/fl  
Dhh+**

**P2RY14  
Nf1 fl/fl  
Dhh+**

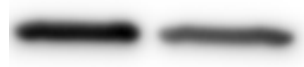

**-b-actin**

Supplement: Source data 2. [file elife-73511-data2.zip › Source data 2/Figure 4C sciatic b-actin-source data 3.pdf]

Nf1 fl/fl Dhh+  
Vehicle

Nf1 fl/fl Dhh+  
Rolipram 5mg/kg

pPKA  
substrate  
RRXS\*/T\*

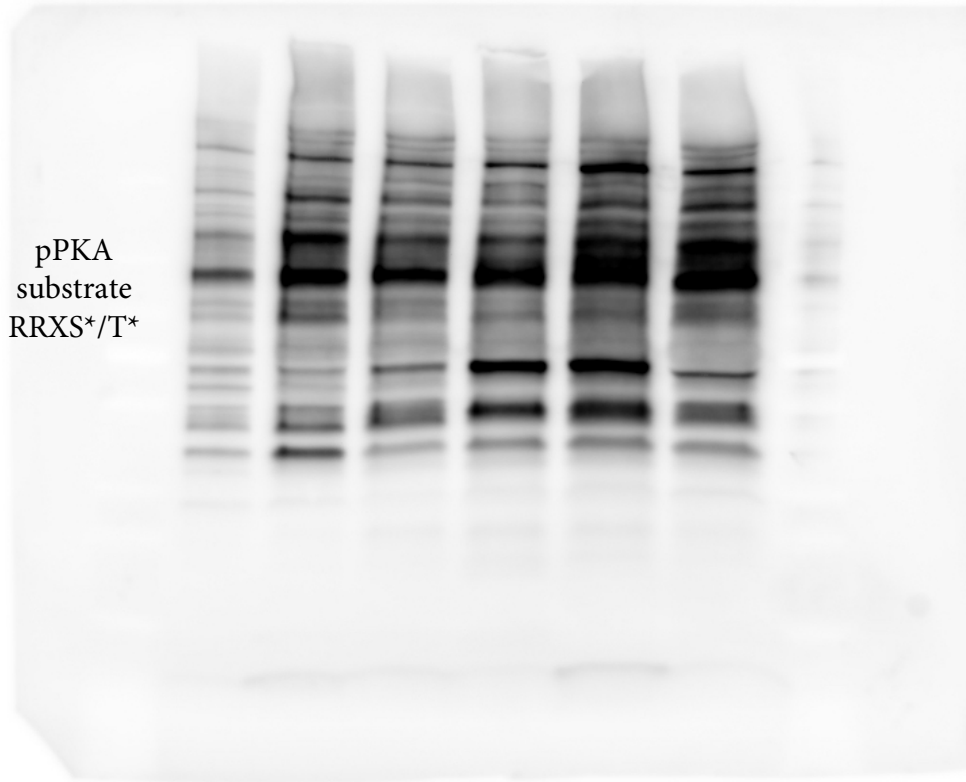

Supplement: Source data 2. [file elife-73511-data2.zip › Source data 2/Figure 6B-pPKA-source data 2.pdf]

8/27/08  
Actin

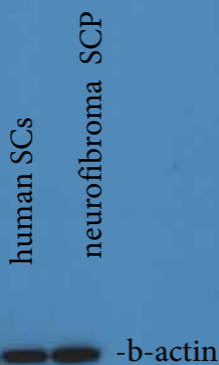

Supplement: Source data 2. [file elife-73511-data2.zip › Source data 2/figure 1B b-actin-source data 2.pdf]

1 lamda

|                  |                                                                                     |                                                                                     |                                                                                     |                                                                                     |                                                                                     |                                                                                     |                                                                                     |                                                                                      |                                                                                       |                                                                                       |                                                                                       |                                                                                       |
|------------------|-------------------------------------------------------------------------------------|-------------------------------------------------------------------------------------|-------------------------------------------------------------------------------------|-------------------------------------------------------------------------------------|-------------------------------------------------------------------------------------|-------------------------------------------------------------------------------------|-------------------------------------------------------------------------------------|--------------------------------------------------------------------------------------|---------------------------------------------------------------------------------------|---------------------------------------------------------------------------------------|---------------------------------------------------------------------------------------|---------------------------------------------------------------------------------------|
| UDP              | -                                                                                   | -                                                                                   | -                                                                                   | -                                                                                   | -                                                                                   | +                                                                                   | +                                                                                   | +                                                                                    | -                                                                                     | +                                                                                     | +                                                                                     | +                                                                                     |
| P2RY14 inhibitor | -                                                                                   | -                                                                                   | -                                                                                   | -                                                                                   | +                                                                                   | -                                                                                   | +                                                                                   | +                                                                                    | +                                                                                     | -                                                                                     | -                                                                                     | -                                                                                     |
| IBMX             | +                                                                                   | -                                                                                   | +                                                                                   | -                                                                                   | +                                                                                   | +                                                                                   | +                                                                                   | +                                                                                    | +                                                                                     | +                                                                                     | +                                                                                     | +                                                                                     |
| b-actin-         | 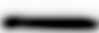 | 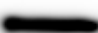 | 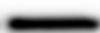 | 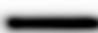 | 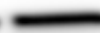 | 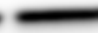 | 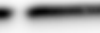 | 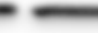 | 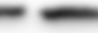 | 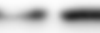 | 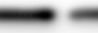 | 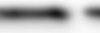 |

Supplement: Source data 2. [file elife-73511-data2.zip › Source data 2/Figure 3C 1 lambda b-actin-source data 2.pdf]

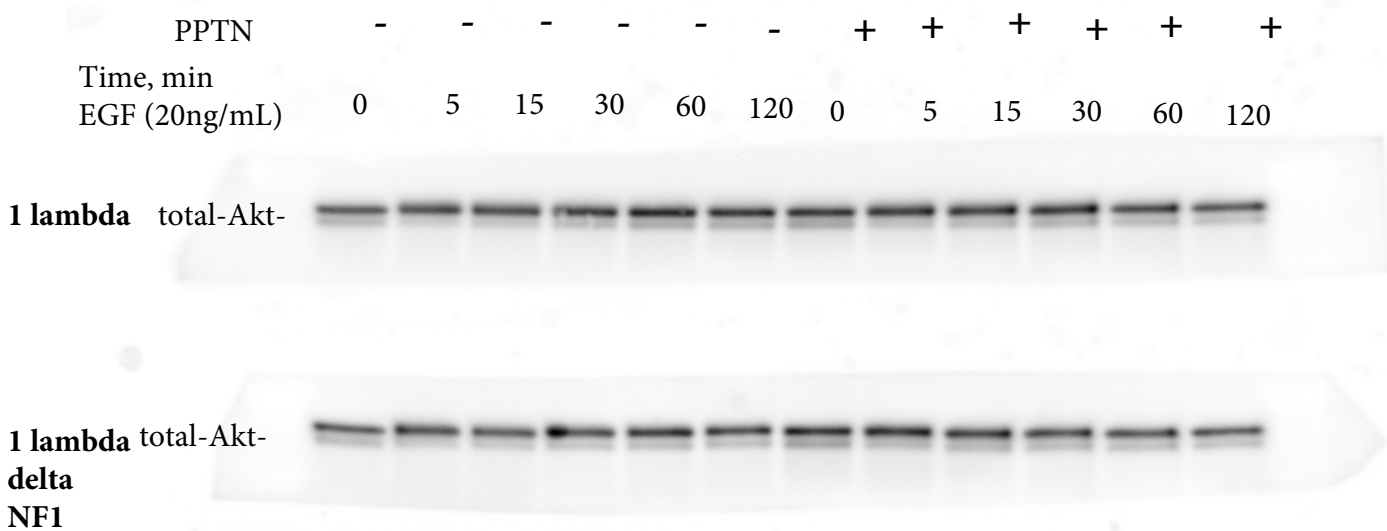

Supplement: Source data 2. [file elife-73511-data2.zip › Source data 2/Figure 3D 1 lambda top and delta NF bottom total AKT-source data 2.pdf]
