# Supplementary material for: P2RY14 cAMP signaling regulates Schwann cell precursor self-renewal, proliferation, and nerve tumor initiation in a mouse model of neurofibromatosis
Source: eLife. 2022 Mar 21;11:e73511. doi: 10.7554/eLife.73511 (PMC8959601; doi:10.7554/eLife.73511)
Supplement: Source data 2. [file elife-73511-data2.zip › Source data 2/figure 1B P2RY14-source data 2.pdf]

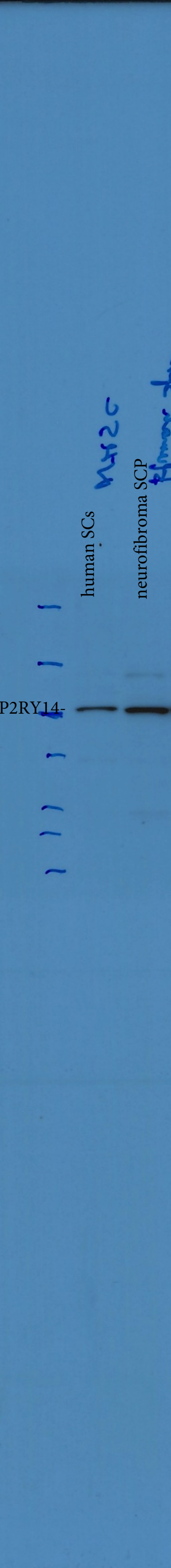
human SCs
neurofibroma SCP
P2RY14-
